# Predicting bacterial infection outcomes using single cell RNA-sequencing analysis of human immune cells

**DOI:** 10.1038/s41467-019-11257-y

**Published:** 2019-07-22

**Authors:** Noa Bossel Ben-Moshe, Shelly Hen-Avivi, Natalia Levitin, Dror Yehezkel, Marije Oosting, Leo A. B. Joosten, Mihai G. Netea, Roi Avraham

**Affiliations:** 1 Department of Biological Regulation, Weizmann Institute of Science, 7610001 Rehovot, Israel; 2 Department of Internal Medicine and Radboud Center for Infectious Diseases, Radboud University Medical Center, 6525 HP Nijmegen, the Netherlands; 3 Department for Genomics & Immunoregulation, Life and Medical Sciences Institute (LIMES), University of Bonn, 53115 Bonn, Germany

**Keywords:** Predictive medicine, RNA sequencing, Bacterial host response, Bacterial infection

## Abstract

Complex interactions between different host immune cell types can determine the outcome of pathogen infections. Advances in single cell RNA-sequencing (scRNA-seq) allow probing of these immune interactions, such as cell-type compositions, which are then interpreted by deconvolution algorithms using bulk RNA-seq measurements. However, not all aspects of immune surveillance are represented by current algorithms. Here, using scRNA-seq of human peripheral blood cells infected with *Salmonella*, we develop a deconvolution algorithm for inferring cell-type specific infection responses from bulk measurements. We apply our dynamic deconvolution algorithm to a cohort of healthy individuals challenged ex vivo with *Salmonella*, and to three cohorts of tuberculosis patients during different stages of disease. We reveal cell-type specific immune responses associated not only with ex vivo infection phenotype but also with clinical disease stage. We propose that our approach provides a predictive power to identify risk for disease, and human infection outcomes.

## Abstract (web summary)

Complex interactions between different host immune cell types can determine the outcome of pathogen infections. Here, Avraham and colleagues present a deconvolution algorithm that uses single-cell RNA and bulk RNA sequencing measurements of pathogen-infected cells to predict disease risk outcomes.

## Introduction

The host-pathogen interface involves, on the host side, hierarchic responses and defense strategies of multiple immune cell types. These different immune cells form a complex network of communications that maintain an orchestrated and dynamic immune response, aimed at eliminating invading agents. To date, significant molecular knowledge was gained by focusing on the interaction of pathogens with innate immune cells, mostly macrophages^1–5^ or dendritic cells^3,6,7^. This assumes that the key to infection outcome is provided by one cell type of interest. Even within these seemingly homogenous populations, emerging technologies that permit accurate profiling of individual cells have revealed significant complexity of innate immune cells^8–10^, and their interactions with bacterial pathogens^11–13^. Adding to this complexity of dynamic activation are the diverse simultaneous facets of immune cells identity; a taxonomy of discrete cell types with continuous transitions of cell activation states. It is exactly this complexity of an intact immune system that is needed to mount an efficient immune response against invading agents.

Recent advances in single-cell RNA-seq (scRNA-seq) allow breakdown of complex tissues and host compartments into cell types and their relevance in health and disease^14^. This approach has revolutionized our ability to understand the immune system in unprecedented level of details, e.g., in processes such as hematopoiesis^9,15,16^, tumorigenesis^17–19^, the lymph node compartment^20^, and responses to microbial ligands^8,21^. This ever-increasing wealth of data has spawned analytical methods, such as computational deconvolution methodologies, that predict detailed cell-type compositions from bulk gene expression data^17,22,23^. However, these algorithms are based on a-priori knowledge or specific datasets^24,25^, which are inherent to the experimental systems. Despite this classification of immune cell types, we currently lack a functional understanding of the dynamic immune response across multiple cell types that allow clearance of invading pathogens.

Peripheral blood mononuclear cells (PBMCs), which contain lymphocytes, natural killer (NK) cells, monocytes, and dendritic cells (DC), provide a window to the complexity of the human immune system which can be assessed in clinical contexts, both in health and in pathological cases^26^. Importantly, bloodborne and other pathogens can reach the blood in cases of systemic infection^27–29^, and cells from the blood are known to migrate to the site of infection^30,31^. Indeed, blood immune cells are used to investigate the effect of different pathogens on the immune system^32–37^. However, these studies are usually based on bulk measurements of PBMCs that overlook the underlying complexity of diverse cell types. How the immune system integrates signals and orchestrates responses from different cell types and how inter-individual variation in these cell types is translated to differences in infection outcome are fundamental to our understanding of human infection biology.

In the current study, we develop a dynamic deconvolution algorithm that captures the two aspects of immune surveillance from bulk measurements: cell-type composition and the dynamics of cell-type specific infection response. To expose the infection-induced states of immune cells, we infect them ex vivo with *Salmonella enterica* serovar Typhimurium (*Salmonella*). We perform scRNA-seq and characterize gene signatures that captures infection-induced states for each cell type and sub-type. We use this mapping to train our deconvolution algorithm, allowing us to predict cell-type specific immune responses from bulk RNA-seq measurements. To demonstrate the algorithm functional utility, we recruit groups of healthy individuals, measure their PBMCs response to ex vivo infection with *Salmonella* and apply our dynamic deconvolution algorithm. We also apply our algorithm to bulk RNA-seq data from cohorts of tuberculosis (TB) patients during different stages of disease. Importantly, we reveal cell-type specific immune responses associated not only with ex vivo infection outcomes but also with clinical disease stage. We offer that our approach provides a predictive power to identify risk factors for human infectious disease.

## Results

### Immune response of human PBMCs to *Salmonella* infection

**Fig. 1:**
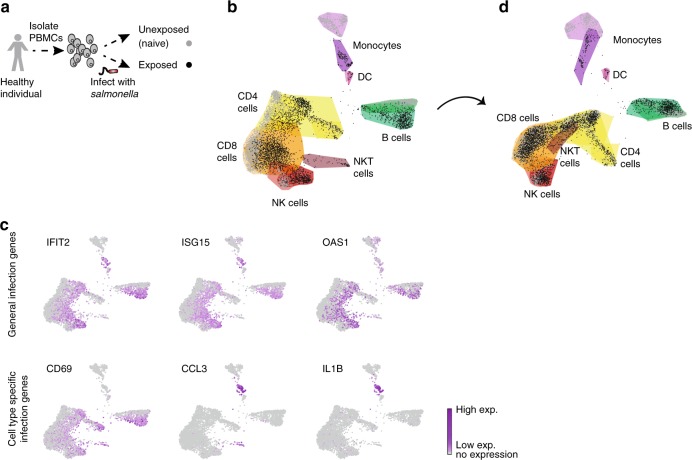
scRNA-seq analysis of human PBMCs before and after ex vivo *Salmonella* infection. **a** Overview of the scRNA-seq experiment: PBMCs were isolated from a blood sample of a healthy individual and were infected ex vivo with *Salmonella* (exposed), or remained unexposed (naïve). Overall ~7000 cells were sequenced using 10x genomics Chromium. **b** Visualization of the scRNA-seq data using forced layout on a two-dimensional space by k-nearest neighbor (KNN)-graph (*k* = 20; naive cells (gray) and exposed cells (black)). K-means clustering of the cells revealed the seven main cell types: NK cells (red), CD8 T cells (orange), CD4 T cells (yellow), NKT cells (brown), B cells (green), monocytes (purple), and dendritic cells (DC; pink), as inferred using cluster-specific genes and marker genes expression (see Supplementary Data 1 and Supplementary Fig. 3d). Colored contours represent cells which belong to the same cell type in each sample (see also Supplementary Fig. 3a–c for complete KNN-graph with edges and clusters). **c** Expression levels of representative genes from the infection signature (see methods and Supplementary Fig. 5). Top: general infection genes which are upregulated following *Salmonella* infection in all exposed cells, and bottom: cell-type specific infection genes. Gene expression is shown using the same layout as in **b**, with the nodes colored by the indicated gene expression in each cell (see colorbar). **d** KNN-graph (*k* = 20) of the scRNA-seq data after removal of the global infection signature eliminated the separation between naïve and exposed cells for all cell types, except for the monocytes, which contain intracellular bacteria. Colors and contours are the same as in **b** (see also Supplementary Fig. 3e–g)

To characterize the dynamics of the host–pathogen interface in a physiological setting that encompass the complex interactions between different immune cell types, we used a model of ex vivo infection of PBMCs with *Salmonella*. We isolated PBMCs from a blood sample of a healthy individual, and infected them ex vivo with *Salmonella*. scRNA-seq was measured for the unexposed (naïve) and exposed cells 4 h after infection, to obtain an unbiased marker-free decomposition of the repertoire of immune cell types and sub-types before and after infection (Fig. 1a, see Supplementary Fig. 1 for experimental design). Although, most of the exposed cells do not contain internalized bacteria, they still respond to the infection. Using flow cytometry and RFP-expressing *Salmonella*, we detected a total of 3% infected cells, with ~90% of the monocytes containing intracellular bacteria (Supplementary Fig. 2). Overall, 7000 cells were analyzed (3515 naïve and 3485 exposed cells). To map the repertoire of immune cell types, we first clustered the cells using k-means clustering, separately for the naïve and exposed cells (Supplementary Fig. 3a–c), and then interpreted clusters identity using cluster-specific genes and known marker genes expression levels^38–45^ (Supplementary Data 1 and Supplementary Fig. 3d). We mapped cells to 7 main cell types: NK, NKT, CD8 T cells, CD4 T cells, B cells, monocytes, and dendritic cells (Fig. 1b). Within each cell-type cluster, we observed a clear separation between naïve and exposed cells (gray vs. black nodes in Fig. 1b and Supplementary Fig. 3a). Noteworthy, monocytes generated two separate groups of naïve and exposed cells, which might be an indication of their unique capacity to contain intracellular bacteria. NKT cells were observed as a distinct cluster only after infection. These changes in monocytes and NKT cells prompted us to experimentally confirm their assigned gene signatures before and after infection. We sorted each population (CD3^+^ CD56^+^ NKT and CD14^+^ monocytes) from naïve and exposed PBMCs and analyzed their transcriptome using bulk RNA-seq. We confirmed that the bulk RNA-seq expression of these sorted populations is highly correlated with the average expression of each cell type in the single-cell data (Supplementary Fig. 4). Further partitioning of the cells into cell sub-types was accomplished using a graph based approach of community detection Louvain on the KNN-graph (see methods), done separately for the naïve and exposed samples. Using this analysis we could further classify 31 cell sub-types in the naïve sample and 29 in the exposed sample (see Supplementary Data 2).

**Fig. 2:**
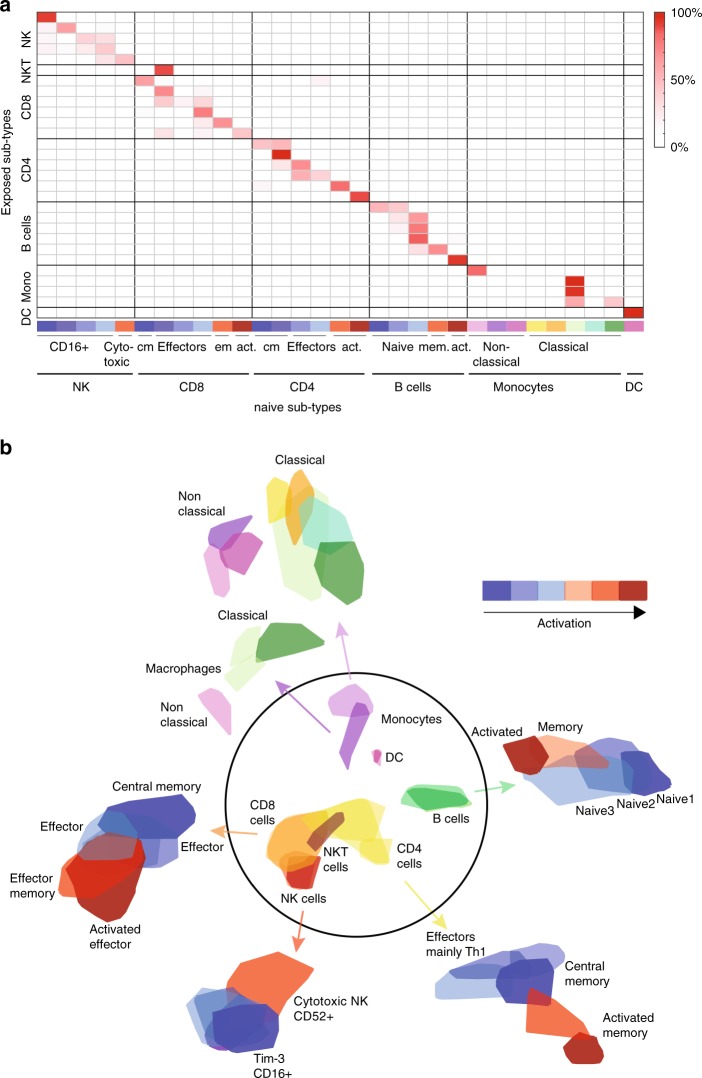
Characterization of human PBMCs intrinsic sub-types before and after *Salmonella* infection. **a** Classification of exposed cells inferred from the cell sub-types of the naive cells using KNN-classification. The connectivity matrix entries are the calculated percentage of cells from each sub-type of the exposed sample (*y*-axis) that were classified to each sub-type of the naive sample (*x*-axis) (see colorbar to the right corresponding to the matrix entries). Cell sub-types identity was inferred from the differentially expressed genes that uniquely characterize each sub-type (Supplementary Fig. 7; color code of sub-types from blue to red represents activation state, found already at steady-state, regardless of the infection response). **b** Graph-based clustering revealed the repertoire of immune sub-types before and after infection (intrinsic fingerprints). The middle circle presents the contours of the cell types based on the KNN-graph from Fig. 1d, and for each cell type further partition into sub-types is shown. Contours are drawn for both the naive and exposed samples together as classified by the KNN-classification in **a**, without the infection genes (colorbar represents activation state from blue to red, which exists already at steady-state, regardless of the infection response). The monocytes were colored based on the different sub-types before and after infection to allow association between the exposed sub-types to the naive sub-types (see also Supplementary Fig. 7 for visualization of the naive and exposed cells relative to the contours and the differentially expressed genes which defines each sub-type)

To characterize the infection dynamics of each cell type and sub-type, we performed an analysis allowing us to trace between the origin of each exposed sub-types in their matching naïve sample. We first curated a global infection signature of 309 genes that were significantly upregulated following *Salmonella* infection, with varying degree of specificity to a certain cell type (Fig. 1c and Supplementary Fig. 5). GO-term enrichment analysis revealed that these 309 genes were indeed significantly enriched for infection terms such as defense response to virus, type I interferon signaling pathway, inflammatory response etc. (Supplementary Data 3). We then removed these genes, eliminating the separation between naïve and exposed cells for all cell types, except for the monocytes which contained intracellular bacteria (Fig. 1d and Supplementary Fig. 3e–g). We then overlaid the exposed cells on top of the centroid of the naïve cells, and classified the exposed cells using KNN-classification, by the sub-types of the naïve sample (Fig. 2a). This connectivity matrix represents the entire repertoire of PBMC sub-types before and after infection. The connection between the cells represents the intrinsic fingerprints which are the inherent characteristic of the cells regardless of the infection axes. Importantly, this connectivity allows us to then infer the infection-induced state of each sub-type, describing the dynamics of the immune cells following infection. By this we separate the scRNA-seq data into two layers, one being the cell-type intrinsic properties which are shared between the naïve and exposed cells (Fig. 2b), and the other a layer of dynamic immune response to infection, exclusive to the exposed cells. For the intrinsic properties, we curated the genes which significantly differentiate between various cell types (Supplementary Fig. 6a), and between sub-types for each cell type (Supplementary Fig. 7). This revealed a range of activation states in the sub-types within each cell type, which exist in PBMCs at steady-state, regardless of infection response (see activation colorbar, Fig. 2b). For example, in B cells, we identified three sub-types of naïve cells, one of memory cells, and another of activated B cells^46^, all of which exist both in naïve and exposed samples, regardless of their response to infection. For the NK cells, their multidimensional projection into two-dimensions preserved mainly the differences between the cytotoxic NK and all other sub-types. Another interesting observation is that after infection with *Salmonella* we observe only three sub-types of monocytes out of the 8 naive sub-types, two of the three resembling M1 polarized macrophages^13^. Loss of some of these monocyte sub-types that are not observed in the exposed sample might indicate a cell-type specific programmed cell death after *Salmonella* infection^47–49^. Finally, we identified that NKT cells originated as a distinct group from a specific sub-type of CD8 T cells, in agreement with the expression profile of the sorted naïve NKT cells (Fig. 2a and Supplementary Fig. 4a). Overall, the resulting gene signature matrices provide a unique immune cell-type fingerprint which we now utilize to bioinformatically predict similar breakdown of PBMCs immune responses from bulk measurements.

### scRNA-seq based deconvolution to model immune responses

**Fig. 3:**
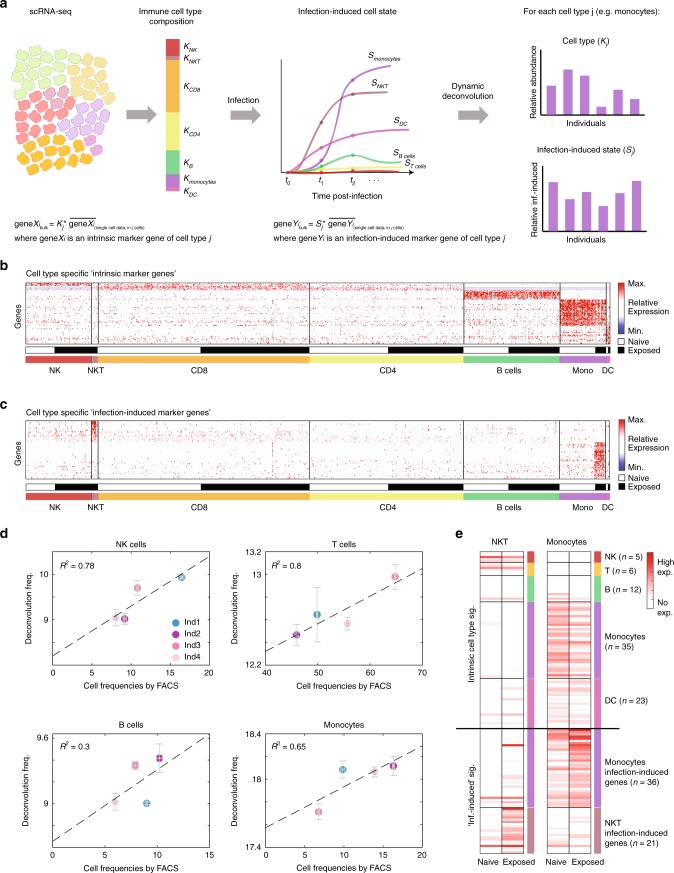
scRNA-seq based dynamic deconvolution to infer cell-type composition and infection-induced states. **a** Illustration of the dynamic deconvolution approach: transformation of the scRNA-seq data into two properties that can be inferred from bulk measurements - immune cell-type composition and infection-induced cell state. Cell-type composition is represented as a one-dimensional vector, where *k*_*j*_ is the number of cells from a specific cell type *j*. The infection-induced cell state (*S*_*j*_) is represented as the induction of cell-type specific genes following infection. Using our deconvolution algorithm (equations at the bottom, see methods) we infer robust estimators for the relative abundance (*K*_*j*_) and infection-induced state (*S*_*j*_) of each cell type across individuals from bulk RNA-seq measurements, as illustrated on the right. **b** and **c** Reduction of the scRNA-seq data into two sets of genes which represent intrinsic cell-type properties (**b**) and cell-type specific infection-induced states (**c**). Cells are ordered by their cell type (color-coded at the bottom) and cell origin (white for naïve and black for exposed cells); see colorbar for expression levels. **d** Validation of our deconvolution algorithm using FACS experiment. Comparison between the percentages of each cell type as measured by FACS (*x*-axis) to the relative abundance by our deconvolution (*y*-axis). There is a high concordance between the deconvolution prediction and the cellular composition as determined by FACS. Each dot is the mean of 3–4 replicates for the FACS and bulk RNA-seq. Presented also are the standard error (SEM) for the replicates. **e** Validation of the infection-induced signatures in sorted populations. Presented are the expression levels of the intrinsic cell types (from **b**) and infection-induced marker genes (from **c**) in bulk measurements of sorted naïve and exposed NKT cells and monocytes. The NKT infection-induced state is upregulated following infection solely in the exposed NKT cells (left). Similarly, the monocytes cell-type signature is expressed exclusively in naïve and exposed monocytes, and the monocytes infection-induced signature is upregulated following infection exclusively in the exposed monocytes (right). Each sample is the mean of 2–4 technical replicates; cell-type signatures are color-coded (*n* denotes the number of genes in each signature)

Using the knowledge we gained from the scRNA-seq data, we constructed a fingerprint of PBMC types and infection-induced cell states (Supplementary Figs. 6a and 5b). We now aimed to deconvolute the same resolution from bulk measurements of complex mixture of PBMCs. While current deconvolution methodologies infer cell-type composition, here we intend to additionally model infection induced-state for each cell type. To achieve this goal, we developed a dynamic deconvolution algorithm for bulk measurements of PBMCs following infection, based on our scRNA-seq data (Fig. 1, Supplementary Figs. 5b and 6a). We first represented the single-cell data as a one-dimensional vector which contains the types and frequencies of PBMCs. Then we represented the infection-induced state of each cell type post-infection (Fig. 3a). Using deconvolution we could predict the vector of relative abundance (*K*_*j*_) and the infection-induced state (*S*_*j*_) of each cell type for any bulk measurements, transforming it into the cell-type resolution of single-cell data. This further allows us to assess the contribution of different complex interactions between cell types to infection outcome, without the need to perform scRNA-seq. To enable deconvolution we applied a specificity filter (see methods) for gene signatures which are expressed exclusively from one cell type, and hence enabled us to recover the cell-type resolution from bulk measurements. We first curated both ‘intrinsic marker genes’ (Fig. 3b), which define uniquely cell types, irrespective of infection; and ‘infection-induced marker genes’ (Fig. 3c), which define the response of a specific cell type to infection (see pipeline in Supplementary Fig. 8a and the method section). For the cell-type specific infection-induced genes, we identified unique signatures only for monocytes and NKT cells, as other infection-induced genes were shared between several cell types (Supplementary Fig. 5b). Next we recovered estimators for cell-type composition (*K*_*j*_) and infection-induced states (*S*_*j*_) from ‘intrinsic marker genes’ and ‘infection-induced marker genes’, respectively. By averaging the set of estimators calculated from each gene alone, we obtain a robust estimation of the immune cell-type composition and infection-induced state within a complex cell mixture of PBMCs (see methods, and Eqs. 3 and 4). Finally, we verified that our marker genes are expressed consistently across all cells belonging to a singular type (Supplementary Fig. 6b) and validated the robustness to all design choices used in our pipeline described above (Supplementary Fig. 8). Using a simulation on synthetic compositions from single-cell data, we modified each design choice (e.g. FDR level, fold-change threshold and specificity filter)^50,51^ and verified that these iterations did not changed the algorithm performance (Supplementary Fig. 8d and Supplementary Data 4–6). Next, algorithm reproducibility was evaluated by applying it on public PBMCs single-cell data^38^. By averaging single-cell data, bulk-like samples with heterogeneous cellular composition were generated (Supplementary Fig. 9a). The inferred compositions by our algorithm were accurate with high *r*-squared values (*R*^2^ > 0.95 for all cell types, Supplementary Fig. 9b), supporting its robustness in an independent experiment. To experimentally validate the algorithm performance, we isolated PBMCs from four individuals and simultaneously performed FACS and bulk RNA-seq. High concordance between the cell-type compositions as measured by FACS and our deconvolution was confirmed (Fig. 3d and Supplementary Fig. 2b). As infection-induced cell states cannot be validated via FACS, specificity and reproducibility was experimentally obtained by sorting naïve and exposed monocytes and NKT cells. Indeed the NKT infection-induced signature was expressed specifically from NKT cells after infection, while the monocytes infection-induced signature was exclusive to the sorted exposed monocytes (Fig. 3e and Supplementary Fig. 2c). Last, we verified the reproducibility of the inferred NKT and monocytes infection-induced signatures using an independent scRNA-seq experiment, in a similar setting (Supplementary Fig. 9c). Importantly, using a simulation on this data we generated bulk-like samples with constant cell-type composition and different infection-induced state of the cells, and evaluated the accuracy of the algorithm to infer the infection-induced state of the cells, regardless of their absolute number. The inferred infection-induced states were accurate with high *r*-squared values (Supplementary Fig. 9d). These validations of the robustness, accuracy, and reproducibility of the algorithm, prompted us to apply it to characterize experimentally the association between immune cell types dynamics and infection outcome.

### Dynamic deconvolution of infection among healthy individuals

**Fig. 4:**
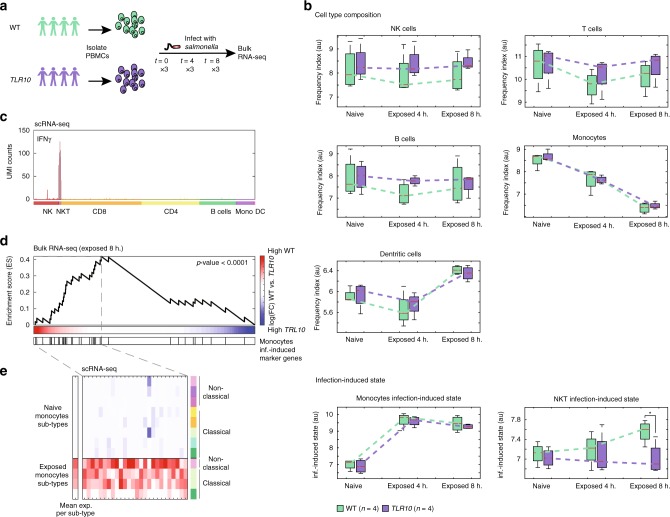
Dynamic deconvolution of immune cell states reveals differences between WT and *TLR10* individuals. **a** Overview of the bulk RNA-seq experiment: isolated PBMCs from blood of eight healthy individuals: WT (green) and *TLR10* (purple), were infected ex vivo with *Salmonella* and bulk RNA-seq was measured before infection (*t* = 0), 4 (*t* = 4), and 8 (*t* = 8) hours post-infection in triplicates. **b** Box-plots of the relative abundance or infection-induced state of each cell type before and 4 or 8 h post-infection in WT vs. *TLR10* uncover significant difference in NKT infection-induced states following infection. The box represents the median and 25–75th percentile, whiskers encompass all data points. *p-value < 0.05, two sample *t*-test. Values are inferred from bulk measurements using our deconvolution algorithm; estimators of cell-type index are in arbitrary units (au). **c** Unique molecular identifier (UMI) counts of IFNγ from each cell by scRNA-seq data revealed production of IFNγ exclusively from NKT cells 4 h post-infection; color-coded cell types are indicated at the bottom. **d** Gene Set Enrichment Analysis (GSEA) of the ‘monocytes infection-induced genes’ in the genes that are higher in WT relative to *TLR10* individuals 8 h post-infection (see methods) reveals partition of the gene signature into two sets which imply differences in sub-types activation following infection. Red to blue bar at the bottom represents the gene expression fold change between WT and *TLR10* individuals (see also colorbar to the right); the black bars below indicate positions of the ‘monocytes infection-induced genes’ in the ordered list of genes. *p*-value is calculated by the maximal Enrichment Score (ES), which also defines the group of enriched genes (all genes to the left of the maximal ES position, i.e. the dashed line). **e** Expression matrix (scRNA-seq data) of the set of ‘monocytes infection-induced genes’ that were enriched in the genes that are higher in WT relative to *TLR10* (genes to the left of the dashed line in **d**). Presented is the mean expression of these genes from each cell sub-type of the naive and exposed monocytes. The left bar represents the mean expression of these genes in each sub-types; monocytes sub-types color-coded as in Fig. 2b

To gain biological insights into the dynamics of human infection, we recruited a cohort of four individuals bearing the wild-type *TLR10* allele (WT) and four individuals bearing a polymorphism in the *TLR1/6/10* locus (*TLR10* N241H (rs11096957), henceforth termed *TLR10)*. *TLR10* polymorphism is associated with altered cytokine production, such as IL-1β and TNF-α when stimulated with bacterial ligands as the Gram-positive Toll-like receptor (TLR)-2 ligands (e.g., Pam3CSK4)^52,53^. TLR10 has been described as the only member of the family to have an inhibitory effect on inflammation, but its ligand specificity and function in different cell types remains unknown. We hypothesized that the altered immune response to bacterial ligands will manifest in differences of distinct immune cell types, which may shed light on *TLR10* function during bacterial infections. We infected ex vivo PBMCs from our group of individuals with *Salmonella*. Bulk RNA-seq was analyzed from each individual’s PBMCs before (naïve; *t* = 0), and 4 and 8 h post-infection (Fig. 4a). Gene-centric analysis identified 1834 genes that were significantly differentially expressed following *Salmonella* infection (1% FDR), but revealed no significant differences between WT and *TLR10* individuals at any time point (Supplementary Fig. 10a–d). To assess whether there are differences between cell-type compositions or infection-induced states between WT and *TLR10* individuals, we applied our algorithm. Interestingly, while we could not detect any significant single gene changes between them, our algorithm found that the infection induced-state of NKT cells was significantly higher in WT vs. *TLR10* individuals 8 h post-infection (*p* = 0.03, two sample *t*-test; Fig. 4b). Importantly, we were able to detect changes in NKT cell dynamics, although their frequency in human PBMC is very low (~1%)^54^. Of note, we found that NKT cells are the only cells which produce IFNγ 4-h post-infection based on our scRNA-seq data (Fig. 4c). In light of this result, we specifically analyzed IFNγ expression, and found no significant difference between WT vs. *TLR10* individuals following *Salmonella* infection (*p* = 0.53 and *p* = 0.21 at 4 and 8 h post-infection, two sample *t*-test; Supplementary Fig. 10e). This exemplifies the advantage of cell-centric perspective over gene-centric analysis and the robustness of our defined infection-induced states.

IFNγ is a major cytokine in host defense against intracellular infection through macrophage activation^55,56^. According to our deconvolution algorithm, we revealed that while significant reduction in monocytes following infection was detected (*p* < 0.005, paired *t*-test; Fig. 4b), as observed also in the scRNA-seq data (Fig. 1b and Fig. 2a), there was a significant corresponding elevation in their infection-induced cell state (*p* < 0.005; Fig. 4b). The advantage of our algorithm is that it allows to model independently the cell proportion and infection-induced state within the same cell type. We hypothesized that there should be a significant difference in monocytes activation in WT vs. *TLR10* individuals due to differences in NKT infection-induced state. To infer variation in monocyte infection-induced states, we performed Gene Set Enrichment Analysis (GSEA) of the ‘monocytes infection-induced marker genes’ in genes that are expressed higher in WT individuals relative to *TLR10* individuals. Indeed, this analysis revealed a bimodal distribution of these genes: significant enrichment of one set in genes which were expressed higher in the WT relative to *TLR10*, while the other set was expressed lower (8 h post-infection, *p* < 0.0001; Fig. 4d). We propose that this partition implies two different monocyte subsets, one of which we suggest is activated by IFNγ mainly in WT individuals. To test this, we examined the expression levels of genes from the ‘monocytes infection-induced marker genes’ which were significantly enriched in the genes that are higher in the WT individuals (Fig. 4d). Indeed, this group of genes showed different expression pattern in the monocyte sub-types, with the highest expression in non-classical monocytes sub-type (Fig. 4e).

Thus, using our dynamic deconvolution algorithm we propose higher infection-induced state of NKT cells in WT compared to *TLR10* individuals. We then wanted to experimentally support cell–cell signaling between NKT cells, IFNγ secretion, and activation following infection of a monocyte subset in WT individuals as suggested by the dynamic deconvolution, and link it to overall infection outcome between WT and *TLR10* individuals.

### Cell–cell signaling affect intracellular bacterial survival

**Fig. 5:**
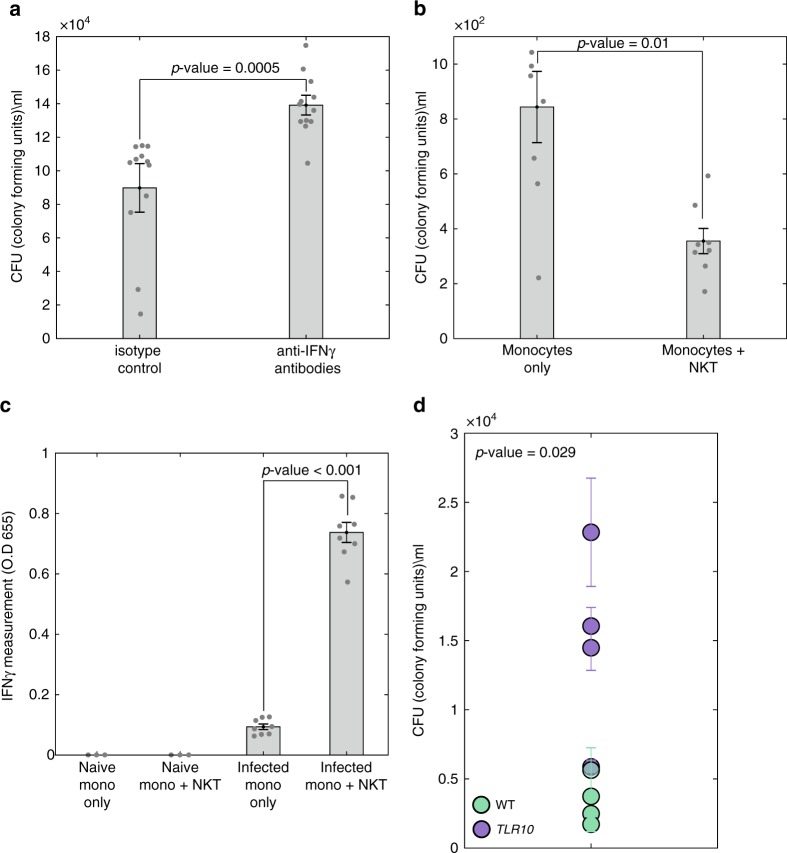
Differences in cell–cell signaling is associated with different bacterial control in WT and *TLR10* individuals. **a** Isolated PBMCs from a WT individual were infected ex vivo with *Salmonella* in the presence of isotype control or anti-IFNγ neutralizing antibodies. Intracellular bacterial growth was determined by CFU 8 h post-infection. Data are presented as bar chart with the average of three independent experiments with four replicates and SEM, all data points are presented by dots. Blocking IFNγ in WT individual increases bacterial load; statistical significance was determined using Friedman’s test, *p*-value is indicated. **b** Intracellular bacteria number was determined by CFU 8 h after ex vivo *Salmonella* infection of monocytes alone or co-culture of monocytes and NKT cells. Data are presented as bar chart with mean and SEM of eight replicates; data points are presented by dots. Co-culture of monocytes with NKT cells provided better control of intracellular bacterial infection relative to monocytes alone; statistical significance was determined using the unpaired Mann–Whitney *U* test, *p*-value is indicated in the figure. **c** Secreted IFNγ levels from monocytes alone or co-culture of monocytes and NKT cells were measured before and after ex vivo *Salmonella* infection. Data are presented as bar chart with mean and SEM of eight replicates; data points are presented by dots. Co-culture of monocytes and NKT cells secreted significantly higher levels of IFNγ relative to monocytes alone; statistical significance was determined using the unpaired Mann–Whitney *U* test, *p*-value is indicated. **d** Isolated PBMCs from eight individuals (WT in green and *TLR10* in purple) were infected ex vivo with *Salmonella*. Intracellular bacterial growth was determined by CFU 8 h post-infection. Data are presented as mean and SEM of three replicates. *TLR10* individuals exhibit higher bacterial load than WT individuals; statistical significance was determined using the unpaired Mann–Whitney *U* test, *p*-value is indicated

To further indicate the role of NKT-monocyte signaling through IFNγ in infection control, we used anti-IFNγ neutralizing antibodies to block IFNγ, and measured its effect on intracellular bacterial survival within PBMCs. By blocking IFNγ in WT individual, an increase in bacterial load was observed by Colony Forming Units (CFU) assay (*p* = 0.0005, Friedman’s test; Fig. 5a). To directly validate signaling between NKT cells and monocytes through IFNγ, we isolated monocytes and NKT cells from PBMCs and infected either monocytes alone or co-culture of monocytes with NKT cells. We found that co-culture of monocytes with NKT cells provided better control of intracellular bacteria relative to monocytes alone (*p* = 0.01, unpaired Mann–Whitney *U* test; Fig. 5b). Furthermore, we detected higher secretion of IFNγ from co-culture of monocytes with the NKT cells relative to monocytes alone (*p* < 0.001, unpaired Mann–Whitney *U* test; Fig. 5c). These results suggest that NKT-monocyte signaling, which is more evident in WT compared to *TLR10* individuals, also provides better control of bacterial infection in WT individuals.

To further validate the link between higher NKT infection-induced state, IFNγ and infection control, we infected PBMCs from these eight individuals with *Salmonella* (four WT and four TLR10 individuals) and measured intracellular bacterial load by CFU. In line with our observation of lower NKT infection-induced state in *TLR10* individuals, they had significantly higher bacterial load 8-h after infection relative to WT individuals (*p* = 0.03, unpaired Mann–Whitney *U* test; Fig. 5d). We further investigated PBMCs from a WT individual and a *TLR10* individual using imaging flow cytometry (ImageStream). ImageStream analysis independently indicated that *TLR10* individual had higher percentage of infected cells relative to WT (Supplementary Fig. 11). Thus, we propose a functional link between the observed NKT infection-induced state and IFNγ secretion to differences in intracellular bacterial control between healthy individuals. We then sought to test if application of dynamic deconvolution to data obtained from a TB patient cohort could functionally predict infection outcomes in a clinical setting of human disease.

### Deconvolution of TB patients predicts disease progression

**Fig. 6:**
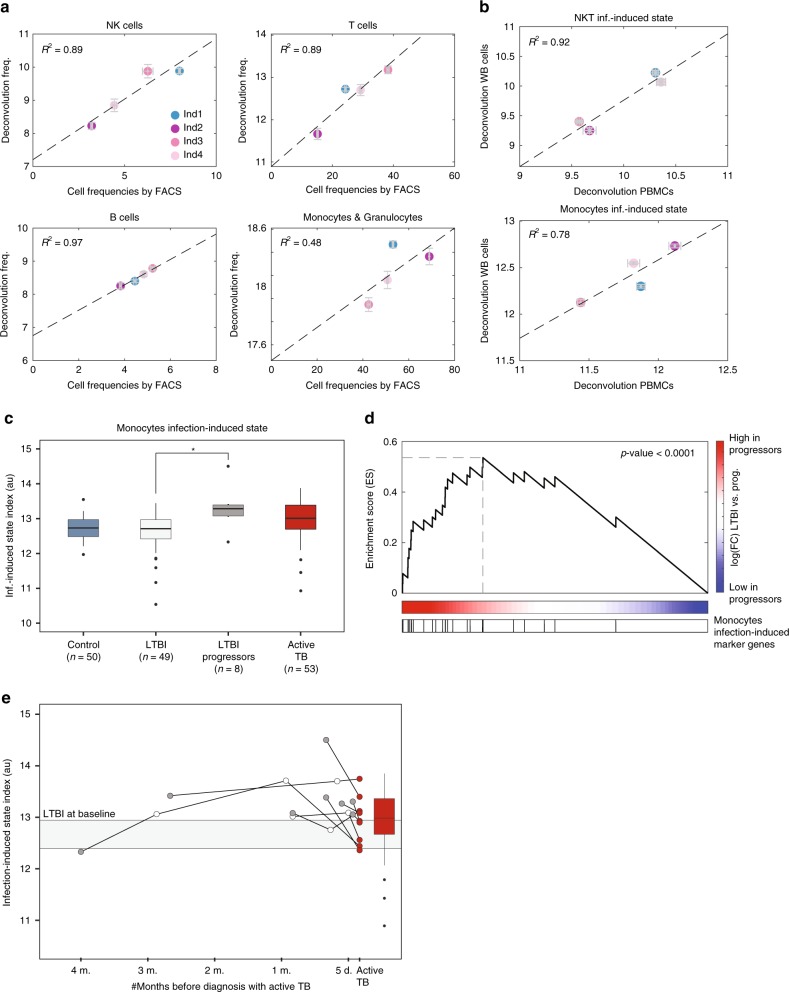
Dynamic deconvolution of the monocytes infection-induced state captures TB progression. **a** Algorithm performance evaluation on WB samples. Comparison between the percentages of each cell type measured by FACS (*x*-axis) to the relative abundance as inferred by our deconvolution algorithm (*y*-axis) for four individuals. There is a high concordance between the deconvolution prediction and the FACS for all cell type, except for the monocytes. Presented is the mean of 3–4 replicates and SEM. *R*-squared values are indicated. **b** Comparison of NKT and monocytes infection-induced states as inferred by our algorithm from matched PBMCs (*x*-axis) and WB samples (*y*-axis) from four individuals. Presented is the mean of four replicates and SEM. There is a high concordance between the infection-induced states as measured from matched PBMCs and WB samples. **c** Box-plot of the monocytes infection-induced state (in arbitrary units- au) of control individuals (blue), LTBI individuals who remained healthy (light gray), LTBI individuals who developed active TB (progressors; dark gray) and active TB patients (red) uncover significant difference between LTBI who remained healthy vs. progressors, before signs of active disease. The box represents the median and 25–75^th^ percentile, whiskers encompass interquartile range. *p-value < 0.05, two sample *t*-test. **d** GSEA of the monocytes infection-induced genes in the genes that are expressed higher in progressors relative to LTBI individuals reveal significant enrichment of the entire signature, *p*-value < 0.0001 (GSEA test). Red to blue bar represents fold change between mean expression of each gene in the progressors relative to LTBI individuals, see methods for more information. **e** Dynamics of the monocytes infection-induced state during TB progression. Deconvolution of the monocytes infection-induced states of nine progressors at several timepoints from baseline (dark gray, as in **c**) until diagnosis of active disease (red) reveals maximal monocytes infection-induced state at the sample preceding the diagnosis of active TB. Box-plots of active TB (red) and LTBI who remained healthy (light gray, shown 25–75^th^ percentile of the samples) are as in **c**, for comparison to the progressors levels. The time before active TB was diagnosis is indicated at the *x*-axis

We examined dynamic deconvolution using three cohorts of TB patient^32,57^, which includes bulk RNA-seq data of whole-blood (WB) cells from active TB patients, individuals with latent infection (LTBI) and control individuals. As our algorithm was trained on scRNA-seq of PBMCs, and WB also contains granulocytes, we first validated the specificity of our cell-type signatures in WB. We extracted WB cells and PBMCs from four individuals and simultaneously performed FACS and bulk RNA-seq analyses. The composition of T, B, and NK cells estimated by our algorithm on WB showed high concordance with the FACS results (Fig. 6a and Supplementary Fig. 2d). The only deviation was of monocytes estimation by the algorithm, which were correlated to monocytes and granulocytes together, instead of monocytes only. Thus, we confirmed the estimation of cell-type composition from WB by our algorithm, and excluded the monocyte cell-type attribute from the analysis of WB. To validate the infection-induced state of the cells, which cannot be directly estimated by FACS, we infected PBMCs and WB samples, performed bulk RNA-seq and analyzed by deconvolution the infection-induced states. The NKT and monocytes infection-induced states were highly reproducible between matched WB and PBMCs samples for all individuals, confirming the accuracy of the algorithm in estimation of infection-induced states from WB samples (Fig. 6b). To further validate that the monocytes infection-induced state from WB is specific to monocytes (and does not capture also granulocytes), we used a public dataset of isolated monocytes and neutrophils from healthy control and active TB patients^58^. The monocytes infection-induced signature were predominantly expressed from isolated monocytes (Supplementary Data 7). Importantly, their expression was negligible in neutrophils (the major cell type that is present in WB and absent from PBMCs^59^). This indicates that the signature is indeed specific to monocyte infection-induced state in the context of WB cells (Supplementary Data 7).

Confirming the utility of deconvolution in WB samples, we applied the algorithm on the TB datasets. Further validating the accuracy of our algorithm, we found significant changes in the cellular composition of the blood of active TB patients relative to LTBI and controls (Supplementary Fig. 12a), as was previously reported for cell-type compositions using other deconvolution algorithms^32^. Importantly, the infection-induced states of the cells, which is a unique feature of our dynamic deconvolution, were significantly different in the blood of active TB patients relative to LTBI and controls, with less NKT cells activation and higher monocyte activation following infection (Supplementary Fig. 12a, b). Among the LTBI individuals, few developed active TB during prospective observations (progressors^32^). Strikingly, the monocytes infection-induced state differentiated between the LTBI who remained healthy and those who developed active TB (*p* = 0.016, two sample *t*-test; Fig. 6c). Importantly, this difference was evident before any symptoms of active disease (at baseline) and was not detected by changes in cell-type composition, only in the infection-induced state of the cells. This suggest a predictive power for the monocytes infection-induced signature to identify LTBI individuals with higher risk to develop active TB. To further corroborate our findings, we extracted a subset of our monocytes infection-induced signature which is expressed only by monocytes and not neutrophils (Supplementary Data 8) and validated that this specific sub-signature is also significantly higher in LTBI progressors compared to LTBI (*p* = 0.027, two sample *t*-test). GSEA of the ‘monocytes infection-induced marker genes’ in the genes expressed higher in the progressors relative to LTBI individuals revealed that unlike in *Salmonella* infection (Fig. 4d, e), the entire signature was significantly enriched (Fig. 6d). Next, we explored the dynamics of TB progression, using a dataset of the progressors individuals that were followed at several timepoints from LTBI at baseline until diagnosis with active TB^32^. Interestingly, the monocytes infection-induced state was significantly higher in the sample preceding diagnosis of active TB in most individuals (*p* = 0.038, paired *t*-test; Fig. 6e). Thus, our deconvolution algorithm provide a predictive power for TB progression by the infection-induced state of the cells, a unique feature that allows us to model infection dynamics with clinical disease.

## Discussion

Bacterial infectious diseases remains a leading cause of mortality globally, with the rate of occurrence of new drug-resistant bacteria significantly outpacing the developmental rate of novel antibiotics. Despite decades of research, we still have limited insights into the host factors that determine infection outcome and the well documented variation between individuals in infection severity^60^. Advances in scRNA-seq can significantly promote our understanding of molecular details that underlie an efficient immune response able to eliminate the pathogen, compared to instances where the immune system fails. In the current study, we applied scRNA-seq to characterize two major aspects of immune surveillance against invading pathogen: the immune cell composition and their responses to infection. However, integration of this knowledge to human infection requires scalability to large cohorts. For this, we developed a dynamic deconvolution algorithm that aims to model, using bulk RNA-seq, the changes in the immune cell types (‘intrinsic marker genes’) and the induced cell states (‘infection-induced marker genes’). Using the deconvolution algorithm, we revealed that *TLR10* polymorphism is associated with attenuated infection-induced state of NKT cells and cell–cell signaling mediated by IFNγ following infection, which in turn influences infection phenotype. Finally, to gain insight into clinical infection outcomes, we applied our algorithm to bulk RNA-seq data from cohorts of TB patients during different stages of disease. Remarkably, our algorithm uncovered differences not only between active TB patients and healthy individuals but could also predict latent individuals that remained healthy to those who progressed to active disease.

The increasing wealth of scRNA-seq data has spawned several deconvolution algorithms allowing the breakdown of a complex bulk measurement to its cell-type constituents^17,22^. However, these algorithms allow estimation only of the cell-type composition, highlighting a blind spot to crucial aspect of the immune surveillance: the infection-induced cell states. To allow dynamic deconvolution, we followed human PBMCs after infection with the enteric pathogen *Salmonella*, a causative agent of enteric human diseases, and characterized a cell-type specific immune response for each cell type in our data. Indeed, bacterial infection is known to induce the activation of multiple cell types and complex cell–cell interactions. Paracrine signaling of IFNγ, IL-22, IL-17 and more, mediated by multiple cell types, such as T cells and NK cells^61^, are important to mount an efficient immune response through activation of antimicrobial processes of cells such as macrophages, and to play a critical role in the control of bacterial pathogens.

PBMCs composition is stable within an individual along time, but between humans, PBMCs composition can vary significantly^26^. This variation might have important implications to the differences in infection outcome between individuals^60^. Thus, we set out to test whether variation in immune cell types and states can account for human variation in infection outcome. For this, we obtained PBMCs from a group of individuals bearing either the WT allele or a polymorphism in *TLR10* gene that modulates the cytokine response. *TLR10* is a part of *TLR1/6/10* locus, which was shown to have the strongest association with alteration in cytokines production in response to various ligands^53^, however the mechanism for this alteration is still unknown. While standard gene-centric analysis revealed no significant changes between the WT and *TLR10* individuals, our dynamic deconvolution algorithm deciphered that NKT infection-induced state is significantly higher in WT vs. *TLR10* individuals. Moreover, we identified a cell–cell signaling circuitry involving IFNγ secretion by NKT cells that activates a specific monocyte subset in WT vs. *TLR10* individuals (Figs. 4d, e and 5). This variation in the infection-induced cell states was translated to differences in infection phenotype between WT and *TLR10* individuals (Fig. 5d). Although the differences in NKT infection-induced states between WT and *TLR10* individuals should be further validated in a larger cohort of individuals, it is tempting to speculate that TLR10 is involved in the process of NKT activation through lipid antigens presented by CD1d in antigen-presenting cells^62^. Notably, the differences between the two groups were associated only with the infection-induced cell states and not with the cell-type composition, which exemplifies not only the advantage of our approach over standard gene-centric analysis but also over other deconvolution algorithms that are not based on dynamic data.

We then applied our algorithm to clinical human disease data sets. Recent efforts are aimed at curating gene signatures from blood immune cells as biomarkers for active TB^32–34^, bacterial meningitis^35^ or acute respiratory infections^36^. In the case of TB, the vast majority of individuals that have been infected with the causative pathogen, *M. tuberculosis*, remain clinically asymptomatic, a state termed as latent TB infection (LTBI). It is therefore considered that effective TB prevention will require diagnosis and early treatment of LTBI that can become active disease. However, none of the current curated gene signatures has provided the resolution of our approach. Having validated the accuracy of our algorithm on WB samples, we applied our deconvolution on bulk RNA-seq datasets that were obtained from longitudinal sampling of TB patients. Strikingly, monocyte infection-induced state differentiated between latent individuals who remained healthy to those who developed active TB (progressors) already at baseline, before any symptoms of active disease were evident (Fig. 6c). Thus, while our algorithm was trained on dynamic data of PBMC infection with *Salmonella*, the cell types and infection-induced states represents also stereotypic immune activation upon perturbation, relevant also to clinical disease with other pathogens. This suggest a predictive power for the monocytes infection-induced signature as a classifier of latent individuals with higher risk to develop active TB. Importantly, using longitudinal data of TB progressors, which allowed modeling of disease progression (Fig. 6e), we detected a decrease in monocyte infection-induced state at the time of active disease diagnosis relative to the preceding sample (Fig. 6e). This finding might implicate our infection-induced signatures to cell-type specific responses that occur during early disease stages.

Our findings emphasize the limitation of current deconvolution algorithms that are focused mainly on inferring cell-type composition at steady-state. We envision that in order to understand infection, our dynamic deconvolution approach can be adopted to other experimental models. It is reasonable to assume that the infection dynamics has characteristics which are global, as the monocyte infection-induced state in the case of *Salmonella* and TB, and characteristics which are pathogen-specific, as the NKT infection-induced state in the case of *Salmonella* infection outcome. We propose that further scRNA-seq experiments with additional pathogens and at additional time-points are needed in order to fully uncover the global and pathogen-specific cell-type immune responses. These can be further used to train dynamic deconvolution to model more refined resolution to clinical disease in different infectious agents.

The ability to associate different infection-induced states and infection outcome has a tremendous potential in preventing and curing infectious disease. Our approach allows identification of latent individuals with high risk to develop active TB disease, and can suggest policies directed at treatment of LTBI, for effective TB prevention. Taken together, our dynamic deconvolution algorithm, functional phenotypic assays and clinical data provide a robust approach to analyze complex interactions between immune cell types and help to uncover important inductive signaling cues during early stages of bacterial infection. We believe that this approach is fundamental to our understanding of host–pathogen interactions, and propose advanced treatment options for better control of bacterial infection.

## Methods

### Isolation and preparation of PBMCs

After obtaining informed consent, venous blood was drawn from the cubital vein of volunteers into 10 ml EDTA Monoject tubes (Medtronic, Dublin). The PBMCs fraction was extracted by density centrifugation of EDTA blood diluted 1:1 in pyrogen-free saline over Ficoll-Paque (Pharmacia Biotech, Uppsala). The PBMCs were washed twice in PBS and suspended in RPMI 1640 medium supplemented with gentamicin (10 mg/mL), l-glutamine (10 mM), and pyruvate (10 mM). The procedure was approved by the scientific ethic committee at the Radboud University Medical Center (NL42561.091.12) and the Weizmann Institutional Review board (25–1). The cells were counted and frozen until used. A day before each experiment, the cells where defrosted, washed with PBS, and suspended in medium (RPMI 1640 with l-Glutamine supplemented with 10% heat inactivated fetal bovine serum and 1 mM sodium pyruvate) and plated on untreated plates. A day after, the cells were collected from the dish. To avoid cell lost, the dish was washed with medium and the remaining cells were added to the collected cells. The cells were then manually counted with trypan blue and normalized so the same amount of cells was used for each individual (1–5×10^5^ cells per replicate, depends on the amount of live cells in the least concentrated sample).

### Isolation of whole blood cells and PBMCs from fresh blood

After obtaining informed consent, venous blood was drawn from the cubital vein of healthy volunteers, then whole blood (WB) cells and PBMCs were isolated immediately as follows: 5 ml of filtered 6% dextran (T-500) in PBS was mixed with 3.5 ml of citrate buffer (25 g Na Citrate, 8 g Citric acid in 500 ml PBS, filtered). Then 8.5 ml of the mixed dextran + citrate buffer were added to 30 ml of fresh blood with gentle mixing, and left standing for 30 min in RT. After the incubation, the upper phase (plasma and WB) was transferred to a new tube, and divided for WB or PBMCs isolation. For WB, left overs of red blood cells were lysed by Red Blood Cell (RBC) lysis buffer (Sigma, 11 814 389 001) according to the manufacturer’s manual. For PBMCs isolation from the same blood samples, the plasma + WBC fraction was further fractionated as described in the method section of isolation and preparation of PBMCs without using antibiotics. The isolated WB and PBMCs were counted with Trypan blue, and 5×10^5^ cells were used per well in a 96-well dish.

### Monocytes and NKT isolation

After PBMCs isolation, monocytes were enriched as follows: 3 ml of 50–70×10^6^ PBMCs/ml were layered over 10 ml hyperosmotic percoll solution [48.5 ml percoll (Sigma, P1644–100ML), 41.5 ml sterile DDW, and 10 ml 1.6 M NaCl solution] before centrifugation at 580×*g* for 15 min at RT. The interface was gently collected, washed by cold PBS and resuspended in medium (RPMI 1640 with l-Glutamine supplemented with 10% heat inactivated fetal bovine serum and 1 mM sodium pyruvate). For NKT enrichment, CD3^+^ CD56^+^ NKT Cell Isolation Kit (Miltenyi Biotec, 130-093-064) was used according to the manufacturer’s manual.

### Ex vivo infection with *Salmonella*

*Salmonella* strains used in this study were derived from the wild-type strain SL1344 containing GFP (pFPV25.1; Addgene) or RFP^63^. Cultures of *Salmonella* were grown in Luria-Bertani (LB) medium at 37 °C for 16 h and used for PBMCs infection at MOI 25 for the exposed cells, and PBS was added to the naive samples. After 30 min of internalization, the cells were washed and suspended with media containing 50 μg/ml gentamicin to eliminate *Salmonella* that were not internalized. The cells were incubated for the time indicated in each experiment at 37 °C in 5% CO_2_ in non-treated cell culture plates. For the scRNA-seq in Supplementary Fig. 9c, d, the infection was done as appears above however with MOI 5.

### Flow cytometry and ImageStream

At the indicated time point after infection, the cells were collected from the plates. The dish was washed with medium and the remaining cells were added to the collected cells. The cells were washed with PBS and suspended with FACS buffer (20% FBS and 1 mm EDTA in PBS), then stained with APC/Cy7 anti-human CD14 antibodies (301820) (Supplementary Fig. 2) or APC/Cy7 anti-human CD14 antibodies (301820), PE/Cy7 anti-human CD19 (302216), APC anti-human CD56 (BD341027), PE anti-human CD3 (300408; Fig. 3e and 6a) for 30 min in 4 °C under dark conditions. After washings the cells were resuspended in FACS buffer, live/dead staining SYTOX blue (S34857) was added and the cells were analyzed FACSAria™ III flow cytometer (BD Biosciences) and Diva software. For all antibodies 1 μl of antibody per 100 μl volume of cells was used, except for APC anti-human CD56 (BD341027) which 5 μl of antibody per 100 μl volume of cells were used. SYTOX blue (S34857) was added according to the manufacturer’s recommended quantity. Monocytes were sorted (Fig. 3d, Supplementary Fig. 4) according to FSC/SSC localization, NKTs were sorted (Fig. 3d, Supplementary Fig. 4) according to CD3^+^ CD56^+^^64^ positive cells. For imaging flow cytometry (ImageStreamX mark II imaging flow-cytometer; Amnis Corp), samples were analyzed by IDEAS 6.2 software. RFP-positive cells were considered infected and RFP-negative uninfected.

### RNA extraction and library preparation for scRNA-seq

Four hours after infection, the cells were washed with PBS, counted with trypan blue, suspended with 0.04% BSA in PBS and directly used for single-cell sequencing by the Chromium Single Cell 3′ Reagent version 2 kit and Chromium Controller (10X Genomics, CA, USA). Library quality and concentration were assessed according to the manufacturer’s instructions. For the scRNA-seq in Supplementary Fig. 9c, d after the wash with PBS the cells were resuspended in 120 μl of 5 mM EDTA and incubated 5 min in RT to detach cells that might have attached the dish. The EDTA was then washed and the protocol was continued as appears above.

### scRNA-seq data preprocessing

The Cell Ranger Single-Cell Software Suite (https://support.10xgenomics.com/single-cell-gene-expression/software/pipelines/latest/what-is-cell-ranger) was used to perform sample demultiplexing, alignment to the genome (GRCh38), barcode assignment for each cell, gene counting by unique molecular identifier (UMI) counts (a random sequence which is essential for correction for individual molecules), and merging of the naïve and exposed samples using cellranger aggr which aggregates multiple GEM well. Overall we sequenced 7000 cells; 3515 cells from the naïve sample, with ~80,500 mean reads per cell, ~1800 median UMI count per cell and ~800 median genes per cells. For the exposed sample we sequenced 3485 cells with ~76,000 mean reads per cell, ~1800 median UMI count per cell, and ~830 median genes per cells.

### scRNA-seq data normalization and gene filtration

Only genes with at least one UMI count detected in at least one cell were used. Data were normalized to a library size factor. Factors were calculated by dividing total UMI counts in each cell to the median of the total UMI counts across all cells. Data were transformed to log10 scale (log10(UMI count + 1)). We filtered out cell cycle and ribosomal genes, and selected the top 5000 most variable genes for further analysis. Variable genes were selected based on fitting of the data to a simple noise model based on the genes mean expression and dispersion (coefficient of variance)^65^. We selected the top 5000 genes that were distant from the fit line (i.e. most variable over the noise model).

### scRNA-seq data analysis

Principal component analysis (PCA) was performed on the top 5000 most variable genes, and the first 20 PCs were used for downstream analysis for k-means clustering and construction of k-nearest neighbor (KNN)-graph, based on Euclidian distance in PC space. The data was first analyzed for each samples alone. For the naïve sample we obtained 10 clusters using k-means clustering and the elbow method (Supplementary Fig. 3b), and nine clusters for the exposed sample (Supplementary Fig. 3c). Clusters identity was inferred using cluster-specific genes; we calculated the expression difference of each gene between the mean expression in the cluster and the median of the rest of the clusters^38^. Genes were ranked based on their expression difference, and we labeled each cluster to a cell type based on the top 100 cluster-specific genes (Supplementary Data 1). We also verified the expression of known marker genes, e.g.: NK (NKG7 and GNLY), NKT (CD3D and NKG7), CD8 T cells (CD3D and CD8A), CD4 T cells (CD3D, LDHB and IL7R), B cells (MS4A1, CD79A and CD79B), monocytes (LYZ and CD14 and/or CD16), and DC (LYZ and CCR7) (see Supplementary Fig. 3d)^39,41–45,66^. Further partition of each cell type into cell sub-types was done using Louvain community detection^67^ on the KNN-graph of each sample (with *k* = 20 or 30 depending on the number of cells obtained in each cell type). The Louvain community detection algorithm identify communities of densely inter connected cells in a way that maximizes modularity. We used the Jaccard similarity to build the KNN-graph, i.e., gave weight for each edge between two cells based on the number of their mutual neighbors, in order to strength connections between cells from the same community. We used an implementation of Louvain method for community detection (http://netwiki.amath.unc.edu/GenLouvain), which optimize modularity-like quality function by iterating the Louvain algorithm until convergence. Overall, we obtained 31 cell sub-types in the naïve cells and 29 in the exposed cells. Visualization of the data was done using force-layout of the KNN-graph.

### Doublets detection in scRNA-seq data

Doublets detection was performed following clustering since the variability in UMI counts between cells arises also from different cell types, and not merely due to doublets. When examining the UMI counts between clusters, there was one cluster in the B cells with higher number of UMIs than all other B cells sub-types. Furthermore, this cluster expressed T cells marker genes (such as CCL5, TRAC, and CD3D) in addition to the B cells markers. Therefore we decided to excluded this cluster from further analysis, since we suspected that these cells are B:T doublets (22 cells from the naïve cells and 21 from the exposed cells). We further corroborated this conclusion by performing doublets simulation: we artificially generated doublets from our cells and repeated the analysis. Our cluster for suspected B:T doublets was indeed located at the same area as the B:T simulated doublets. Moreover, none of the other clusters were located at the area of simulated doublets. With that being said, we cannot exclude existence of doublets from the same cell type.

### Infection signature identification in scRNA-seq data

To identify the genes that are upregulated following infection across the various cell types, we identified all significantly upregulated genes in each cell type post-infection, with 1% FDR and a minimum log-ratio of 0.2 between naïve and exposed cells. Overall we identify 309 genes (union of all upregulated genes from each cell type), some of these were unique to specific cell type, and some were shared among several cell types (Supplementary Fig. 5).

### KNN-classification

We classified the exposed cells into the naïve cell sub-types using KNN-classification in order to identify the origin of each cell sub-type in the exposed cells. The classification was done after the removal of the infection signature, using the knnclassify function in matlab.

### scRNA-seq differentially expressed genes thresholds

We identified 238 genes which are significantly differentially expressed between the various cell types with 1% FDR and a minimal fold change of 1.5-fold. For the cell sub-types, since the differences between the sub-types is less pronounced than the separation between the cell types, we used a more conservative FDR threshold and lower fold-change threshold: 0.001%FDR with a minimum fold change of 1.4-fold. These analyses were done for the intrinsic cell types and sub-types and therefore were performed on the data after removal of the infection signature, for the naïve and exposed cells together.

### Expression matrix normalization

Preceding visualization of the data in expression matrix genes were centered and normalized to a mean of 0 and a standard deviation of 1.

### Cell types and sub-types contours in scRNA-seq analysis

To draw the boundaries of a group of cells (e.g. from the same cell type as in Fig.1b or cell sub-type as in Fig. 2b), we used the boundary function in matlab. Before calculating the boundaries, we excluded outliers with more than three standard deviations from the median.

### Intrinsic cell type maker genes identification in scRNA-seq

We curated gene lists which are significant and specific for each cell type (see Supplementary Fig. 8a). To control for significance, we selected genes that are significantly differentially expressed between the various cell types (1% FDR and a minimum fold change of 1.5-fold; Supplementary Fig. 6a). To control for unique expression in a specific cell type we applied a specificity filter (see details below).

### Cell-type specific infection-induced maker genes (scRNA-seq)

We applied the specificity filter (see below) on the global infection signature (see infection signature identification in scRNA-seq data section and Supplementary Fig. 5b) to curate gene lists which are specific to infection in a certain cell type.

### Specificity filter for marker genes identification

One of the basic features of our deconvolution signatures is their specificity to one cell type. In order to define a gene as a cell-type specific marker gene it should fulfill two thresholds: (1) the average expression of the gene from all cells in the specific cell type is >0.5 (log2 scale), and <0.5 in all other non-relevant cell types (termed exp. threshold); and (2) the sum of the gene expression across each cell types (that is not the specific one) is lower than third of the population (termed specificity threshold). This ensure that not only the average expression of the gene is lowly expressed from all non-relevant cell types, but it is also expressed only from a low fraction of the population.

### Statistical analysis

To identify genes that are significantly differentially expressed between cell types or sub-types we performed ANOVA. FDR levels and minimum fold-change thresholds are indicated in each section.

### Dynamic deconvolution algorithm

We developed a deconvolution algorithm to infer cell-type composition and the dynamics of infection-induced cell state from bulk RNA-seq measurement of a mixture of immune cells (PBMCs) before and after infection. Our main assumption is that the expression of a gene in the bulk is the sum of its expression from each cell in the sample (Eq. 1)

1 $${\mathrm{geneX}}_{\left( {{\mathrm{bulk}}} \right)} = \mathop {\sum }\limits_{i \in {\mathrm{cells}}} {\mathrm{geneX}}_{\left( {{\mathrm{sc}}\;{\mathrm{data}};i} \right)}$$

where geneX_(bulk)_ is the expression of a gene *x* in the bulk, geneX_(sc data; i)_ is the expression of a gene *x* from cell *i* in the single-cell (sc) data.

We can reduce this equation for marker genes that expressed specifically from one cell type *j* (Eq. 2).

2 $${\mathrm{geneX}}_{\left( {{\mathrm{bulk}}} \right)} = K_j^\ast {\mathrm{geneX}}_{\left( {{\mathrm{sc}}\;{\mathrm{data}};j} \right)}$$

where *j* is a specific cell type (e.g. NK, B cells etc.), *K*_*j*_ is the number of cells from cell type *j* in the single-cell data, and geneX_(sc data; *j*)_ is the mean expression of gene *x* in the single-cell data, which expressed exclusively from cell type *j*.

As the major factor for successful deconvolution is accurate gene signatures^22^, we curated gene lists from our single-cell data which expressed specifically from one cell type and describe the cell-type relative abundance and infection-induced cell state (Fig. 3 and Supplementary Fig. 8a). Using combination of the expression levels of these marker genes from the bulk and the scRNA-seq data, we deconvoluted an estimator for the relative abundance (K) and infection-induced state (S) of each cell type in the bulk RNA-seq measurements (Eqs. 3 and 4).

From Eq. 2 we can extract *K*_*j*_ for a specific marker gene:

3 $$K_j = \frac{{{\mathrm{geneX}}_{\left( {{\mathrm{bulk}}} \right)}}}{{{\mathrm{geneX}}_{\left( {{\mathrm{sc}}\;{\mathrm{data}};j} \right)}}}$$

where *j* is a specific cell type and gene *X* is an intrinsic marker gene for cell type *j*.

We calculate the *K*_*j*_ estimator for each intrinsic marker gene, and by averaging all estimators of a specific cell type *j* we get a robust estimation of the relative abundance of cell type *j* ($\widehat {K_j}$)

Similarly, we can calculate *S*_*j*_ by each infection-induced marker gene:

4 $$S_j = \frac{{{\mathrm{geneY}}_{\left( {{\mathrm{bulk}}} \right)}}}{{{\mathrm{geneY}}_{\left( {{\mathrm{sc}}\;{\mathrm{data}};j} \right)}}}$$

where *j* is a specific cell type and gene *Y* is an infection-induced marker gene for cell type *j*.

We calculate the *S*_*j*_ estimator for each infection-induced gene, and by averaging all estimators of a specific cell type *j* we get a robust estimation of the infection-induced state of cell type *j* ($\widehat {S_j}$).

Using these cell-type estimators we can compare between the relative abundance or dynamic of infection-induced state of each cell type from different individuals as measured from a mixture of cells.

### Robustness analysis of the dynamic deconvolution algorithm

We validated that the performance of our algorithm are not dependent on the design choices used in the scRNA-seq analysis. Supplementary Figure 8a details the entire pipeline of the scRNA-seq analysis and all design choices used to generate the deconvolution signatures; we modified each design choice (D.C.) and evaluated its effect on the algorithm’s signatures and performance:

1. D.C. (1) – clustering: to validate the robustness and consistency of the clustering we sub-sampled randomly 1500 cells out of the 3500 naïve cells 10 times, applied similar clustering method and compared the resulting clusters (see Supplementary Fig. 8b for representative sub-sampling of the data vs. the original clustering of the entire data).
2. D.C. (2) – FDR level and fold-change threshold to define the global infection signature: we modified the FDR level to range between 0.1 and 10%, and the fold-change threshold to range between 0.15–0.3 log ratio (see Supplementary Data 4 for the original thresholds and implications of the modified thresholds).
3. D.C. (3) – FDR level and fold-change threshold to define the genes which are significantly differentially expressed between the various cell types: we modified the FDR level to range between 0.01 and 10%, and the fold-change threshold to range between 1.2- and 1.7-fold (see Supplementary Data 5 for the original thresholds and implications of the modified thresholds).
4. D.C. (4) – exp. threshold and specificity threshold for the specificity filter: we modified the – exp. threshold to range between 0.3 and 0.6, and the specificity threshold to range between 0.1 and 0.5 (see the method section specificity filter for marker genes identification for explanation about these thresholds, and Supplementary Data 6 for implications of the modified thresholds).

We evaluated the implications of the modified design choices on the algorithm performance using simulation on our scRNA-seq data: we generated bulk-like expression data from our single-cell data by averaging the expression data of each gene across all cells (simulated bulk expression). We generated five such synthetics mixes of cells to get bulk-like samples with different compositions (for each composition we randomly selected 1000 cells out of the data; mix1-mix5 in Supplementary Fig. 8c). We than applied our deconvolution algorithm with the original signatures, and the modified signatures from the modification of each design choice, to evaluate its performance (Supplementary Figure 8d).

To evaluate the reproducibility of our deconvolution algorithm in an independent sample, we performed a simulation on public scRNA-seq data of PBMCs^38^. We generated five synthetic mixes of bulk-like samples with different compositions from this data (see the section above for more information), and applied our deconvolution algorithm to assess its robustness in an independent individual (Supplementary Fig. 9a, b).

To evaluate the reproducibility of the infection-induced states inferred by our deconvolution algorithm in an independent individual, we performed additional scRNA-seq experiment. PBMCs were isolated from an independent individual, ex vivo infected with *Salmonella* and sequenced using the 10X Chromium (see details in the method sections above: Isolation and preparation of PBMCs, ex vivo infection with *Salmonella*, and RNA extraction and library preparation for scRNA-seq). We sequenced 5986 cells with ~42,000 mean read per cell, ~2800 median UMI count per cell, and ~900 median genes per cell. Using a simulation on this data we generated 20 synthetic mixes of bulk-like samples with constant cell-type composition and different infection-induced state of the cells. To generate the constant cell-type composition we randomly selected for each synthetic mix 2000 cells with constant number of NKT cells (50 cells), constant number of monocytes (500 cells), and the rest of the cells were selected randomly from all other cell types. By preserving the number of NKT cells and monocytes in all mixes, we ensured that the differences are due to the different infection-induced state of the cells, and not derived from different number of cells. For each bulk-like sample we calculated the infection-induced state of the sample based on the single-cell data, and applied our deconvolution algorithm to assess its accuracy in an independent individual (Supplementary Fig. 9d). The infection-induced state of the sample in the single-cell data were calculated by averaging the expression levels of the NKT infection-induced genes in the NKT cells and the monocytes infection-induced genes in the monocytes.

### Limitations and caveats of the algorithm

The algorithm infer the relative abundance of each immune cell type and its infection-induced state, and not the absolute composition, and therefore can be only used to compare between different samples. The algorithm was trained based on the dynamic infection response of PBMCs to a single pathogen - *Salmonella*, which limits the utility of the algorithm when applied to other pathogens or tissue samples. Thus, further validations for the cell-type specific signatures (similar to the validations done for WB in Fig. 6a, b and Supplementary Data 7 and 8), or scRNA-seq data of infected cells with the relevant pathogen is recommended.

### RNA extraction and library preparation for bulk RNA-seq

At the indicated time points after infection the cells were washed with PBS, then suspended with RLT buffer (from RNeasy Mini Kit, Qiagen) + 1% β-mercaptoethanol and kept in −80 °C until further extraction. RNA was then extracted with RNeasy Mini Kit (Qiagen). RNA-seq libraries were prepared according to Cel-seq libraries protocol^68^ with a minor modification: the starting material was purified bulk RNA. The libraries run on Illumina Nextseq instrument with a total coverage of ~600 M reads and a mean of ~7.5 M reads per library for WT and *TLR10* libraries; ~1.3 M reads and a mean of ~270 K reads per library for the sorted monocytes; ~13 M reads and a mean of ~1.6 M reads per library for the sorted NKTs; ~6.5 M reads and a mean of ~210 K reads per library for the PBMCs libraries for deconvolution algorithm validation; and ~6.2 M reads and a mean of ~200 K reads per library for the WB cells libraries for deconvolution algorithm validation.

### Bulk RNA-seq data preprocessing and normalization

The cel-seq pipeline (https://github.com/yanailab/CEL-Seq-pipeline) was used for sample demultiplexing, alignment to the genome (GRCh38), and gene counting. Data were normalized to a library size factor. Factors were calculated by dividing the total number of reads from each sample to the median of the total number of reads across all samples.

### WT and *TLR10* RNA-seq data analysis

Data were transformed to log2 scale, and minimal expression threshold was set to 2. The three replicates of each sample were averaged, except for three samples we excluded due to low coverage (<100 K exonic reads), and therefore for three samples (*TLR10*#1 *t* = 4 h, *TLR10*#2 *t* = 0, and WT#3 *t* = 0), only two replicates were averaged. Preceding Principal Component Analysis (PCA) we selected all expressed genes (minimal threshold of 4.5 and standard deviation ≥0.2), and genes were centered and normalized to a mean of 0 and a standard deviation of 1. Differential expression before and after infection was calculated using paired *t*-test, and between WT and *TLR10* individuals by two sample *t*-test.

### Sorted monocytes and NKT cells RNA-seq analysis

Data were transformed to log2 scale, and a minimal expression threshold was set to 4. The replicates of each sorted population were averaged, and the correlation to the scRNA-seq data were calculated (the correlation was calculated to the mean expression of each cell type in the single-cell data). The deconvolution algorithm was applied on each sample alone, and the mean of all replicates was calculated for each condition (2–4 replicates for each condition).

### Analysis of PBMCs and matched WB cells RNA-seq

Data were transformed to log2 scale, and a minimal expression threshold was set to 2. To validate the infection-induced state signatures, the deconvolution algorithm was applied on each sample alone, and the mean of all replicates was calculated for each condition (3–4 replicates for each condition).

### Sorting Points Into Neighborhood (SPIN)

Unsupervised method for sorting multidimensional data^69^. Iterative algorithm to create optimal ordering such that the distances between the objects are smallest close the diagonal. This allows identification of group of genes that display similar expression profiles over a range of samples.

### GO-terms and KEGG pathway enrichment

GO-terms and KEGG pathways enrichment analysis was performed using DAVID^70,71^, correcting for multiple testing by FDR.

### Gene Set Enrichment Analysis

We ranked the genes based on their fold-change between the two tested conditions. For the WT vs. *TLR10*, for each time point post-infection (4 and 8 h), we calculated the mean expression of each gene in the WT individuals and the *TLR10* individuals, and calculated the fold change between them. We then ordered the genes by their fold-change. GSEA was performed for the enrichment of the ‘monocytes infection-induced marker genes’ in the ranked list (GSEA algorithm, URL:http://www.broadinstitute.org/gsea/). p-values for the enrichment scores were calculated based on the null distribution of enrichment scores obtained by 10,000 random permutations.

### Cohorts of TB patients

We applied our algorithm on three publicly available RNA-seq datasets of TB patients: Berry London GSE107991, Berry South Africa GSE107992 and Leicester GSE107994^32,57^. These cohorts contain whole-blood RNA-seq data of active TB patients, LTBI and control individuals. Leicester dataset contains also longitudinal data on 9 LTBI individuals who developed active disease during the study. Raw data was downloaded and normalized to a library size factor. Data was transformed to log2 scale, and genes below expression threshold (mean expression <4) were filtered out from further analysis. For GSEA see the section above

### Monocytes infection-induced signature in WB samples

The monocytes infection-induced signature was curated from scRNA-seq data of PBMCs. To validate its specificity in WB cells we used a public dataset of sorted monocytes, sorted neutrophils (the major cell type that is present in WB cells and absent from PBMCs), isolated PBMCs and WB cells from active TB patients and healthy control individuals (GSE42832^58^). Normalized data was downloaded and analyzed for the expression of the monocytes infection-induced signature.

### Colony-forming units (CFU)

PBMCs were infected as mentioned above with *Salmonella*-GFP (ampicillin resistant) at MOI 25. Eight hours after infection, the cells were washed with PBS, suspended with 0.1% triton X-100 and pipetted vigorously to lyse the cells, and incubated in room temperature for 10 min. The bacteria were grown on LB + ampicillin 100 mg/L plates in serial dilutions, grown over night in 37 °C and counted with Scan® 500 automatic colony counter. The experiments were done with at least three replicates.

### Anti-IFNγ experiments

PBMCs from a WT individual were incubated with 1.63 μg/ml anti-hIFN-g antibodies (MAB285) or with isotype control (MAB003) for 2 h. The cells were then infected with *Salmonella*-GFP as appear above at MOI 25. After 30 min of internalization the cells were washed and suspended with medium containing antibiotic and 1.63 μg/ml anti-hIFN-g antibodies or isotype control and incubated for 8 h until used for CFU measurement.

### IFNγ measurement

IFNγ levels were detected by HEK-Blue™ IFN-γ Cells (Invivogen, hkb-ifng) according to the manufacturer’s manual. In short, HEK-Blue™ IFN-γ cells were generated by stable transfection of HEK293 cells with the human STAT1 gene and a SEAP reporter gene under the control of an ISG54 promoter fused to four interferon-gamma-activated sites (GAS). HEK-Blue™ IFN-γ are specific for IFN-γ stimulation, and upon stimulation activate the JAK-STAT pathway and subsequently the expression of the reporter gene. SEAP is secreted in the supernatant and is detectable when using QUANTI-Blue™ (SEAP detection medium, rep-qb1).

### Reporting summary

Further information on research design is available in the Nature Research Reporting Summary linked to this article.

## Supplementary information

Supplementary Information

Description of Additional Supplementary Files

Supplementary Data 1

Supplementary Data 2

Supplementary Data 3

Supplementary Data 4

Supplementary Data 5

Supplementary Data 6

Supplementary Data 7

Supplementary Data 8

Reporting Summary

## Data Availability

All RNA-seq data (scRNA-seq and bulk RNA-seq measurements) have been deposited in NCBI’s Gene Expression Omnibus (GEO) under the super-series accession number GSE122084. Public TB datasets used in this study can be found in GEO under the accession numbers: Berry London GSE107991, Berry South Africa GSE107992 and Leicester GSE107994.

## Code availability

The code for our deconvolution algorithm is available at: https://github.com/noabossel/Dynamic-deconvolution-algorithm.
