# Supplementary material for: Predicting bacterial infection outcomes using single cell RNA-sequencing analysis of human immune cells
Source: Nat Commun. 2019 Jul 22;10:3266. doi: 10.1038/s41467-019-11257-y (PMC6646406; doi:10.1038/s41467-019-11257-y)
Supplement: Supplementary file 1 — Supplementary Information [file 41467_2019_11257_MOESM1_ESM.pdf]

Supplementary data

Predicting bacterial infection outcomes using single cell RNA-sequencing analysis of human immune cells

Bossel Ben-Moshe et al.

This PDF file includes:

Supplementary Figures 1-12

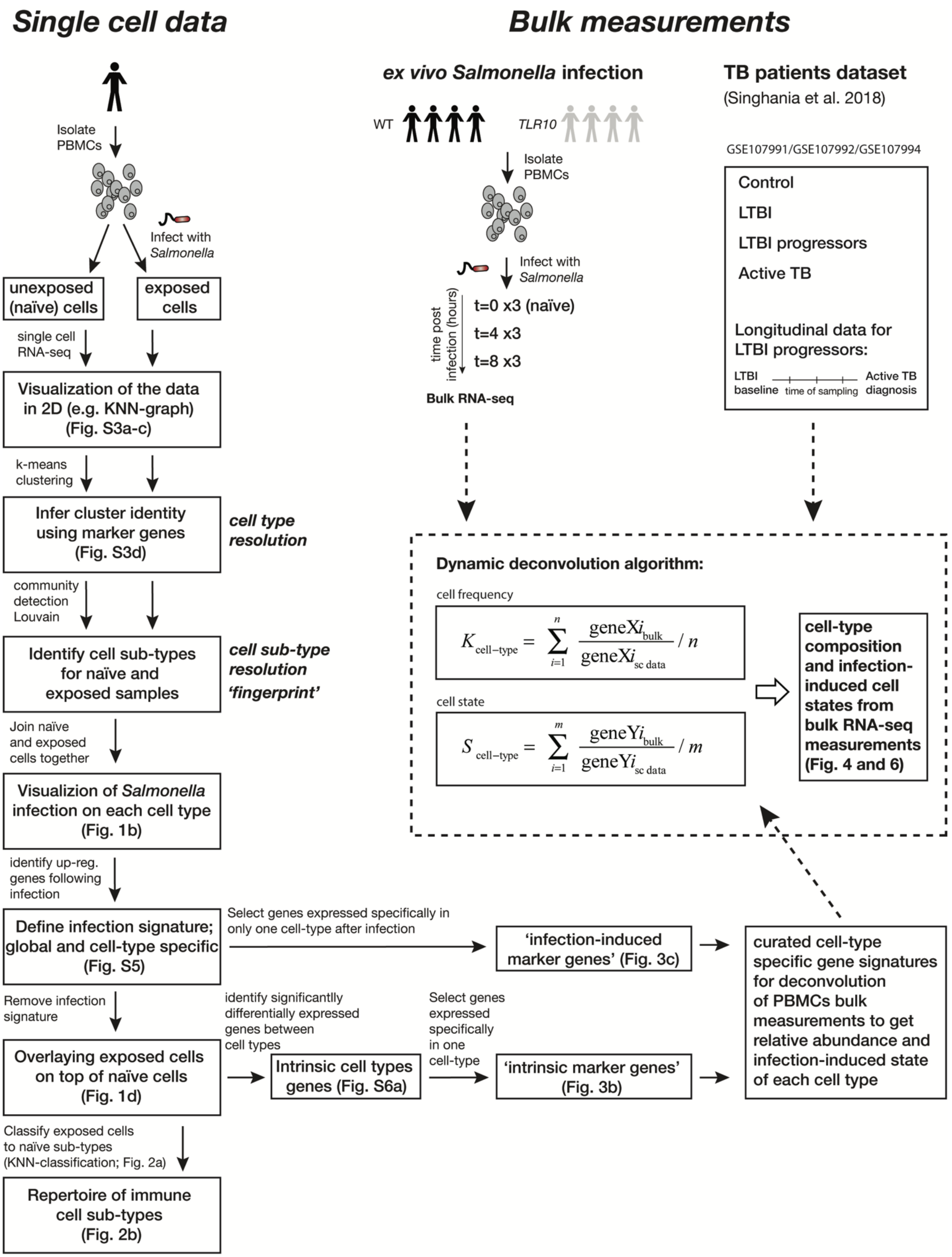

Single cell data
Bulk measurements
Isolate PBMCs
Infect with Salmonella
unexposed (naïve) cells
exposed cells
single cell RNA-seq
Visualization of the data in 2D (e.g. KNN-graph) (Fig. S3a-c)
k-means clustering
Infer cluster identity using marker genes (Fig. S3d)
cell type resolution
community detection Louvain
Identify cell sub-types for naïve and exposed samples
cell sub-type resolution 'fingerprint'
Join naïve and exposed cells together
Visualizion of Salmonella infection on each cell type (Fig. 1b)
identify up-reg. genes following infection
Define infection signature; global and cell-type specific (Fig. S5)
Remove infection signature
Overlaying exposed cells on top of naïve cells (Fig. 1d)
Classify exposed cells to naïve sub-types (KNN-classification; Fig. 2a)
Repertoire of immune cell sub-types (Fig. 2b)
ex vivo Salmonella infection
WT
TLR10
Isolate PBMCs
Infect with Salmonella
time post infection (hours)
t=0 x3 (naïve)
t=4 x3
t=8 x3
Bulk RNA-seq
TB patients dataset
(Singhania et al. 2018)
GSE107991/GSE107992/GSE107994
Control
LTBI
LTBI progressors
Active TB
Longitudinal data for LTBI progressors:
LTBI baseline
time of sampling
Active TB diagnosis
Dynamic deconvolution algorithm:
cell frequency
$K_{\text{cell-type}} = \sum_{i=1}^{n} \frac{\text{geneX}i_{\text{bulk}}}{\text{geneX}i_{\text{sc data}}} / n$
cell state
$S_{\text{cell-type}} = \sum_{i=1}^{m} \frac{\text{geneY}i_{\text{bulk}}}{\text{geneY}i_{\text{sc data}}} / m$
cell-type composition and infection-induced cell states from bulk RNA-seq measurements (Fig. 4 and 6)
Select genes expressed specifically in only one cell-type after infection
'infection-induced marker genes' (Fig. 3c)
identify significantlly differentially expressed genes between cell types
Intrinsic cell types genes (Fig. S6a)
Select genes expressed specifically in one cell-type
'intrinsic marker genes' (Fig. 3b)
curated cell-type specific gene signatures for deconvolution of PBMCs bulk measurements to get relative abundance and infection-induced state of each cell type

Supplementary Figure 1. Overview of the experimental design, bioinformatics analysis and deconvolution algorithm.

Single cell data: PBMCs were isolated from a blood sample of healthy individual and were infected *ex vivo* with *Salmonella* (exposed), or remained unexposed (naïve). Overall ~7000 cells were sequenced using 10x genomics Chromium. scRNA-seq data was visualize on a two-dimensional space using KNN-graph. K-means clustering was performed on each sample alone to obtain cell types, and then further partition of the cell types into sub-types was achieved using graph-based clustering (e.g. Louvain community detection). Next, the naive and exposed scRNA-seq data were joined together, and we identified an infection signature, 309 genes which are significantly upregulated following infection in the various cell types. We removed the infection signature genes from the data to overlay the exposed cells on top of the naive cells, and using KNN-classification associated between the cell sub-types before and after infection. We than defined the intrinsic repertoire of immune cell types and sub-types of human PBMCs before and after *Salmonella* infection (intrinsic cell sub-types 'fingerprints'). We curated cell-type specific gene signatures which characterized the different cell types and their infection-induced cell states to provide a dynamic signatures for the deconvolution algorithm.

Bulk measurements; *ex vivo Salmonella* infection: We isolated PBMCs from blood samples of 8 healthy individuals (4 WT (black) and 4 *TLR10* individuals (gray)). Next, PBMCs were infected *ex vivo* with *Salmonella* and bulk RNA-seq was measured in triplicates from each individual before infection (t=0), 4 hours post-infection (t=4) and 8 hours post-infection (t=8). We characterized the transcriptomic profiles of WT and *TLR10* individuals at early stages of *ex vivo Salmonella* infection.

Bulk measurements; TB patients dataset: To study clinical data of human disease we used publicly available datasets of TB patients (Singhania et al. 2018). The data contains active TB patients, LTBI individuals, LTBI individuals who progress to active disease (progressors) and control individuals. For the progressors individuals a longitudinal data from LTBI baseline to diagnosis of active TB was analyzed to model the dynamics of disease progression.

Deconvolution algorithm: Using our dynamic deconvolution algorithm we analyzed the bulk RNA-seq data into the depth of cell-type composition and infection-induced cell states. This resolution revealed cell-type specific immune responses associated with *ex vivo* infection phenotype and importantly with clinical disease stage.

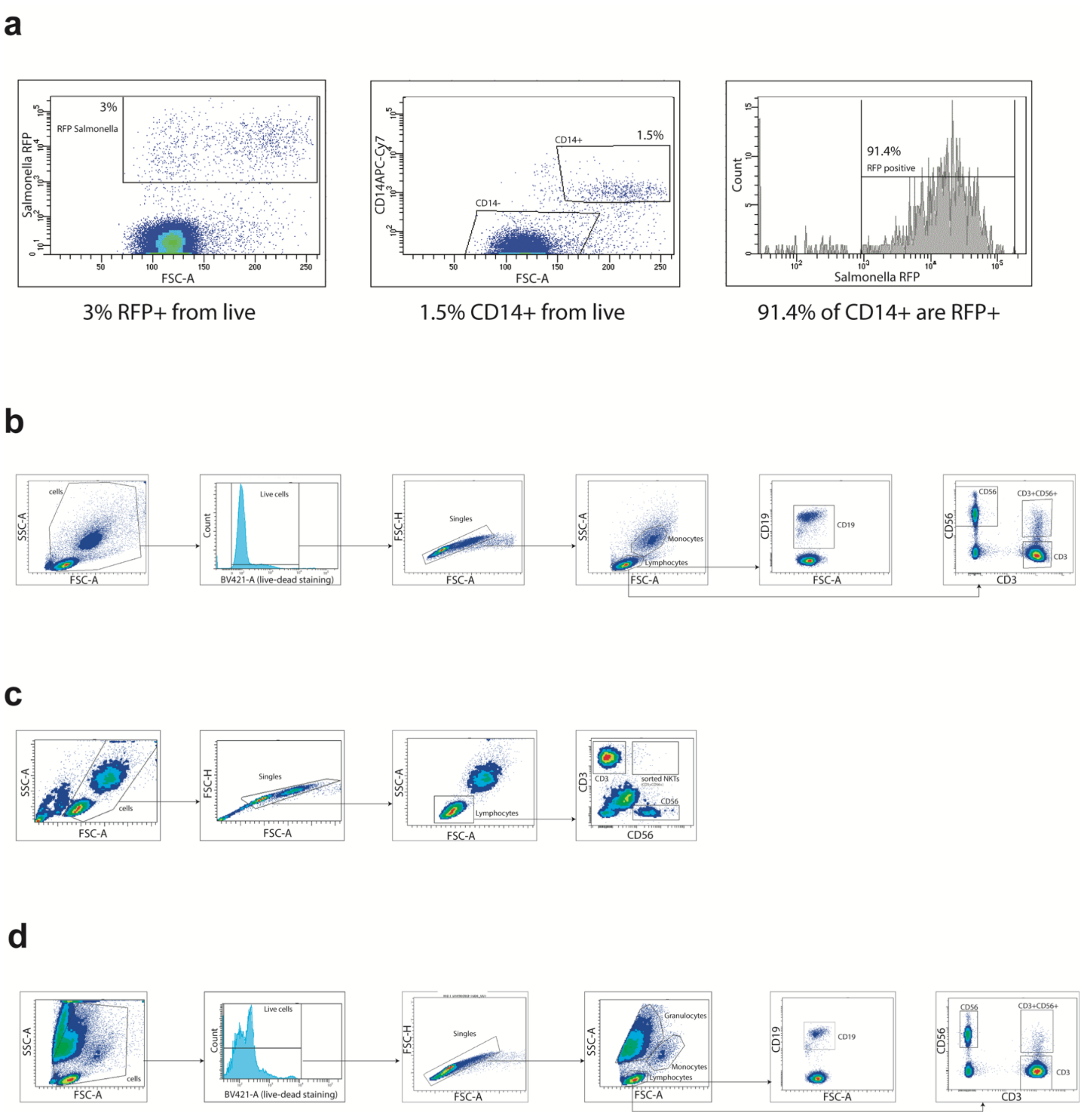

Supplementary Figure 2. Flow cytometry analysis and gating strategies.

a Flow cytometry analysis of *ex vivo Salmonella* infection of human PBMCs. Isolated PBMCs from WT individual were infected *ex vivo* with RFP Salmonella. FACS gating strategies of infected cells (by RFP) and monocytes (CD14) revealed low infection percentage in PBMCs (3%), with high infection rate among the monocytes population (91%). Presented

are the percentages of infected cells from live cells, monocyte cells from live cell and *Salmonella* infected monocytes. **b-d** Gating strategy used for cell analysis and sorting. Cells (5×10^5 cells) were stained as described in the materials and methods section. Examples for gating strategy for PBMCs cell population analysis (**b**), NKT sorting (CD3+CD56+) (**c**) and WB cells analysis (**d**) is shown.

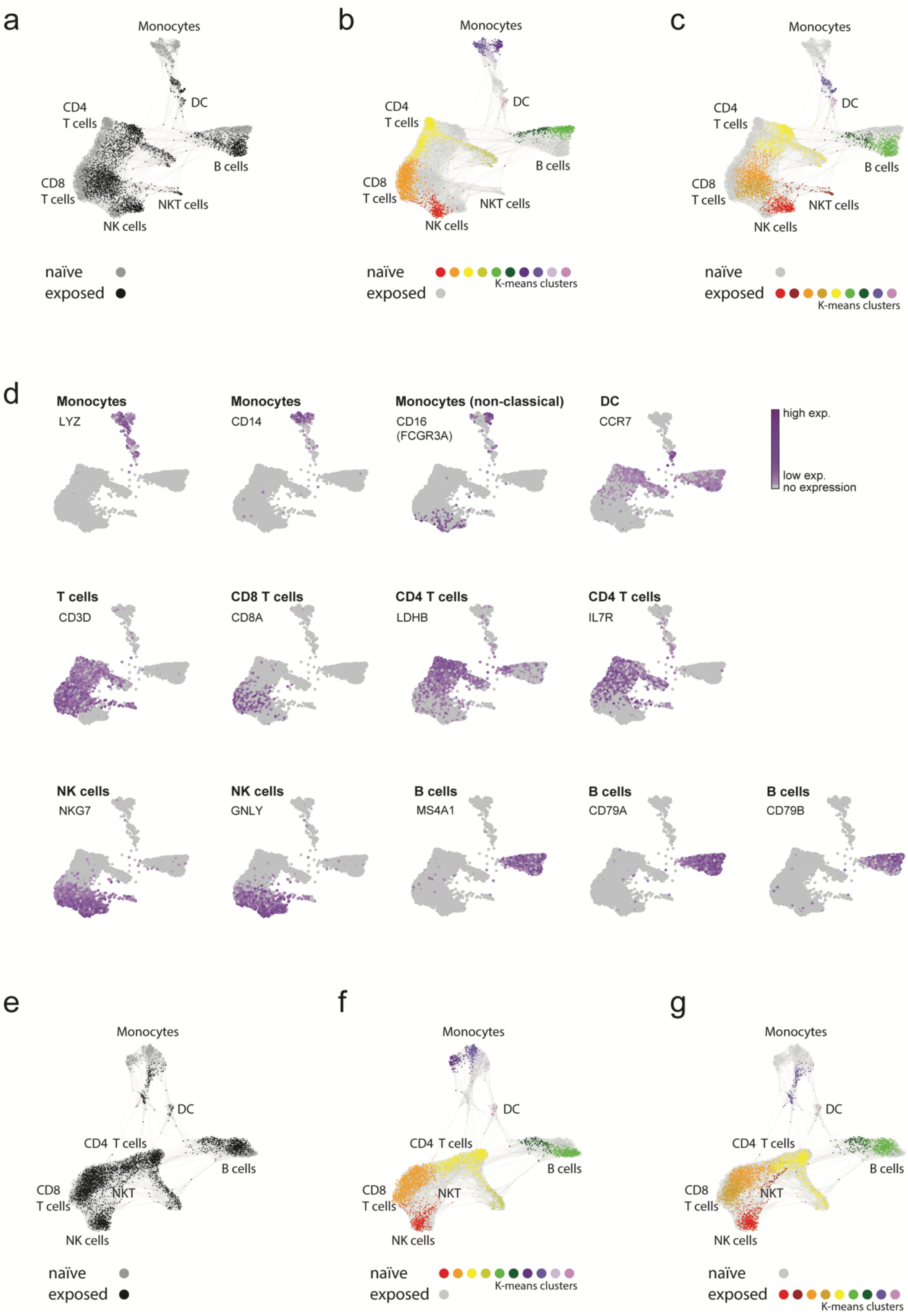
a
Monocytes
DC
CD4 T cells
B cells
CD8 T cells
NKT cells
NK cells
naïve
exposed
b
Monocytes
DC
CD4 T cells
B cells
CD8 T cells
NKT cells
NK cells
naïve
exposed
K-means clusters
c
Monocytes
DC
CD4 T cells
B cells
CD8 T cells
NKT cells
NK cells
naïve
exposed
K-means clusters
d
Monocytes
LYZ
Monocytes
CD14
Monocytes (non-classical)
CD16 (FCGR3A)
DC
CCR7
high exp.
low exp.
no expression
T cells
CD3D
CD8 T cells
CD8A
CD4 T cells
LDHB
CD4 T cells
IL7R
NK cells
NKG7
NK cells
GNLY
B cells
MS4A1
B cells
CD79A
B cells
CD79B
e
Monocytes
DC
CD4 T cells
B cells
CD8 T cells
NKT
NK cells
naïve
exposed
f
Monocytes
DC
CD4 T cells
B cells
CD8 T cells
NKT
NK cells
naïve
exposed
K-means clusters
g
Monocytes
DC
CD4 T cells
B cells
CD8 T cells
NKT
NK cells
naïve
exposed
K-means clusters

Supplementary Figure 3. scRNA-seq data of human PBMCs before and after *ex vivo Salmonella* infection.

a Visualization of the scRNA-seq data on a two-dimensional space using forced layout KNN-graph (k=20; same as in fig. 1b). Dots represent immune cells before (gray; 3493 cells) and 4 hours after *ex vivo Salmonella* infection (black; 3464 cells). **b** and **c** KNN-graph as in **a** colored by the k-means clusters. In **b** naïve cells are colored by their k-means clustering (k=10), and exposed cells colored in gray; in **c** naïve cells colored in in gray and exposed cells are colored by their k-means clustering (k=9). **d** KNN-graph as in **a** in which the cells are colored by the gene expression of the indicated representative marker genes, with gray representing no expression and dark purple high expression (see colorbar to the right). **e** KNN-graph (k=20) of the scRNA-seq data after removal of the global infection signature genes from the data (309 genes, see methods; same as in fig. 1c). Each dot is a cell colored by its origin – gray for naive cells and black for exposed cells. **f** and **g** KNN-graph as in **e** colored by the k-means clusters as in **b** and **c**, respectively. See colormap at the bottom.

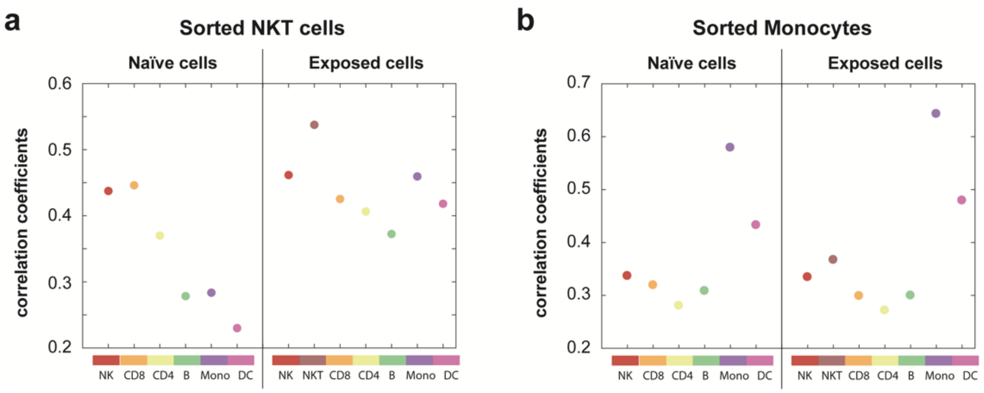

Supplementary Figure 4. Correlation of the sorted NKT cells and sorted monocytes to the single-cell RNA-seq data. Presented are the correlation coefficients values between the expression levels of the sorted naïve/exposed NKT cells (**a**) or monocytes (**b**) to the average expression levels of each cell type in the single-cell data. The sorted exposed NKT cells are with the highest correlation to the exposed NKT cells in the single-cell data (brown dots in **a**), validating our classification of the NKT cells. Similarly, the sorted naïve/exposed monocytes are with the highest correlation to the naïve/exposed monocytes in the single cells data (purple dots in **b**).

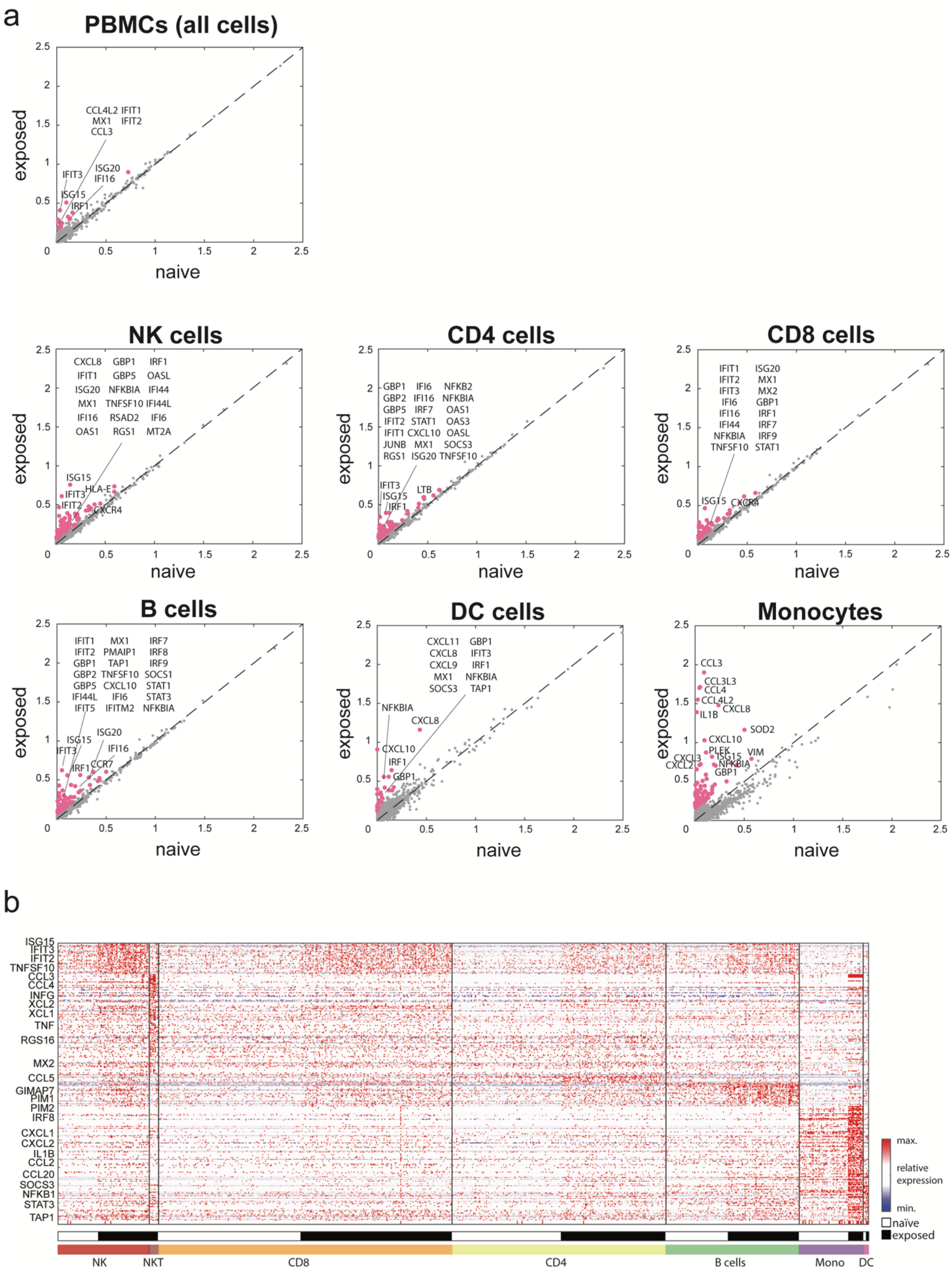
a
PBMCs (all cells)
exposed
naive
CCL4L2 IFIT1
MX1 IFIT2
CCL3
ISG20
IFI16
IFIT3
ISG15
IRF1
NK cells
CXCL8 GBP1 IRF1
IFIT1 GBP5 OASL
ISG20 NFKBIA IFI44
MX1 TNFSF10 IFI44L
IFI16 RSAD2 IFI6
OAS1 RGS1 MT2A
ISG15
HLA-E
IFIT3
IFIT2
CXCR4
CD4 cells
GBP1 IFI6 NFKB2
GBP2 IFI16 NFKBIA
GBP5 IRF7 OAS1
IFIT2 STAT1 OAS3
IFIT1 CXCL10 OASL
JUNB MX1 SOCS3
RGS1 ISG20 TNFSF10
IFIT3
ISG15
IRF1
LTB
CD8 cells
IFIT1 ISG20
IFIT2 MX1
IFIT3 MX2
IFI6 GBP1
IFI16 IRF1
IFI44 IRF7
NFKBIA IRF9
TNFSF10 STAT1
ISG15
CXCR4
B cells
IFIT1 MX1 IRF7
IFIT2 PMAIP1 IRF8
GBP1 TAP1 IRF9
GBP2 TNFSF10 SOCS1
GBP5 CXCL10 STAT1
IFI44L IFI6 STAT3
IFIT5 IFITM2 NFKBIA
ISG20
ISG15
IFIT3
IFI16
IRF1
CCR7
DC cells
CXCL11 GBP1
CXCL8 IFIT3
CXCL9 IRF1
MX1 NFKBIA
SOCS3 TAP1
NFKBIA
CXCL8
CXCL10
IRF1
GBP1
Monocytes
CCL3
CCL3L3
CCL4
CCL4L2
CXCL8
IL1B
SOD2
CXCL10
PLEK
VIM
CXCL3
ISG15
CXCL2
NFKBIA
GBP1
b
ISG15
IFIT3
IFIT2
TNFSF10
CCL3
CCL4
INFG
XCL2
XCL1
TNF
RGS16
MX2
CCL5
GIMAP7
PIM1
PIM2
IRF8
CXCL1
CXCL2
IL1B
CCL2
CCL20
SOCS3
NFKB1
STAT3
TAP1
max.
relative expression
min.
naïve
exposed
NK
NKT
CD8
CD4
B cells
Mono
DC

Supplementary Figure 5. Global infection signature of human PBMCs infected *ex vivo* with *Salmonella*.

a Scatter plots of the mean expression level of each gene across all cells in the PBMCs (top) or in each cell types in the naïve vs. exposed samples. Most genes are across the diagonal (gray dots); genes which are significantly up-regulated following infection (1% FDR and a minimal log-ratio of 0.2) are marked by pink. Names of several known infection genes are indicated in each scatter. **b** Expression matrix of the genes which are significantly up-regulated following infection in all cell types (union of all the genes which are marked by pink in **a**; 309 genes in total). The cells are ordered by their cell type (see colorbar at the bottom of the matrix) and cell origin (white for naive cells and black for exposed cells).

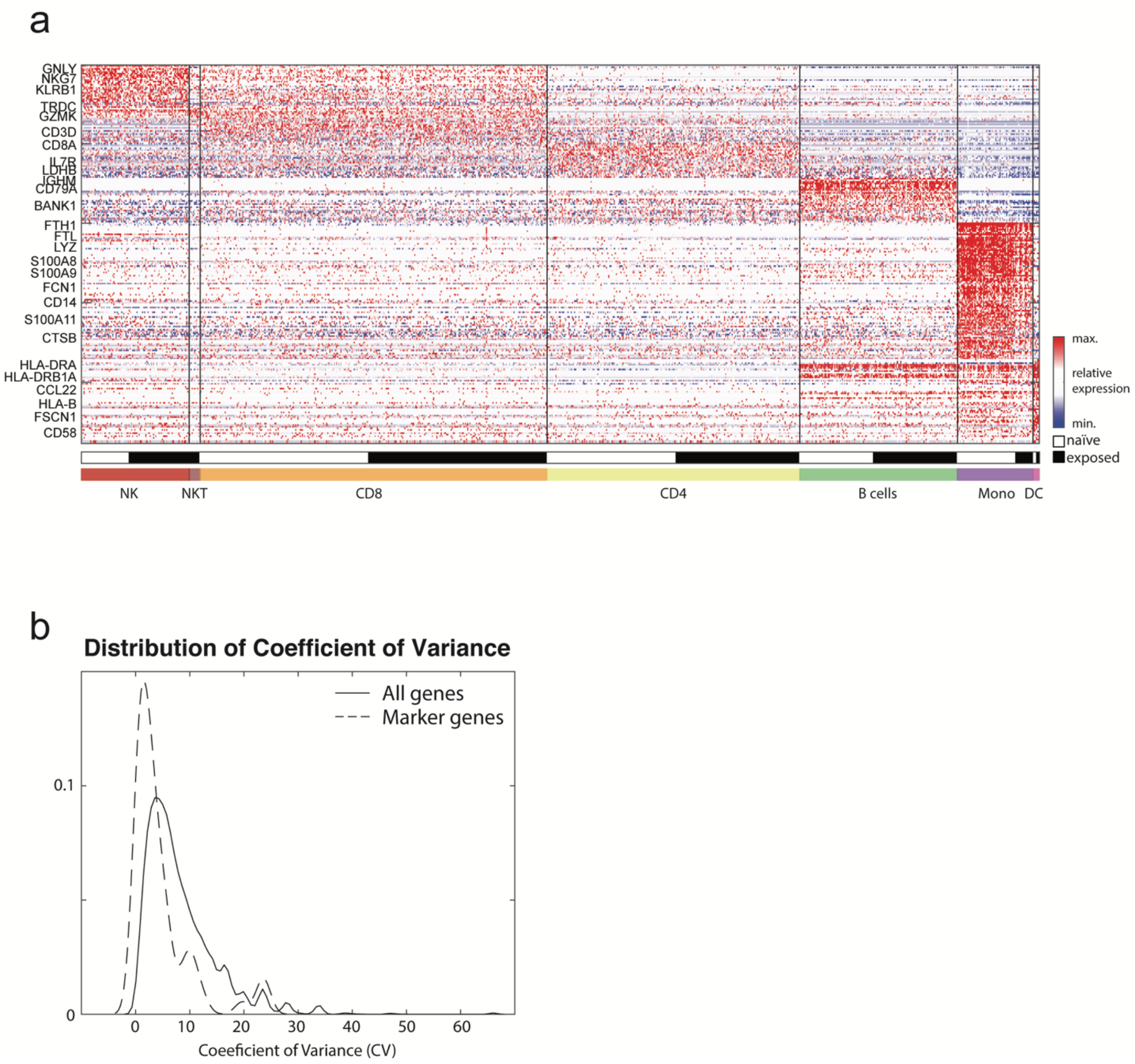

Supplementary Figure 6. Intrinsic cell-type 'fingerprints' of human PBMCs infected *ex vivo* with *Salmonella*.

a Expression matrix of 238 genes which are significantly differentiate between cell types (1% FDR and a minimal fold change of 1.5-fold; without the infection signature) by scRNA-seq data. The cells are ordered by their cell type (see colorbar at the bottom of the matrix) and cell origin (white for naive cells and black for exposed cells). **b** Distribution of the Coefficient of Variance (CV; standard deviation divided by the mean) of all genes (solid line) vs. marker genes (cell-type specific marker genes from fig. 3d drawn in dashed line).

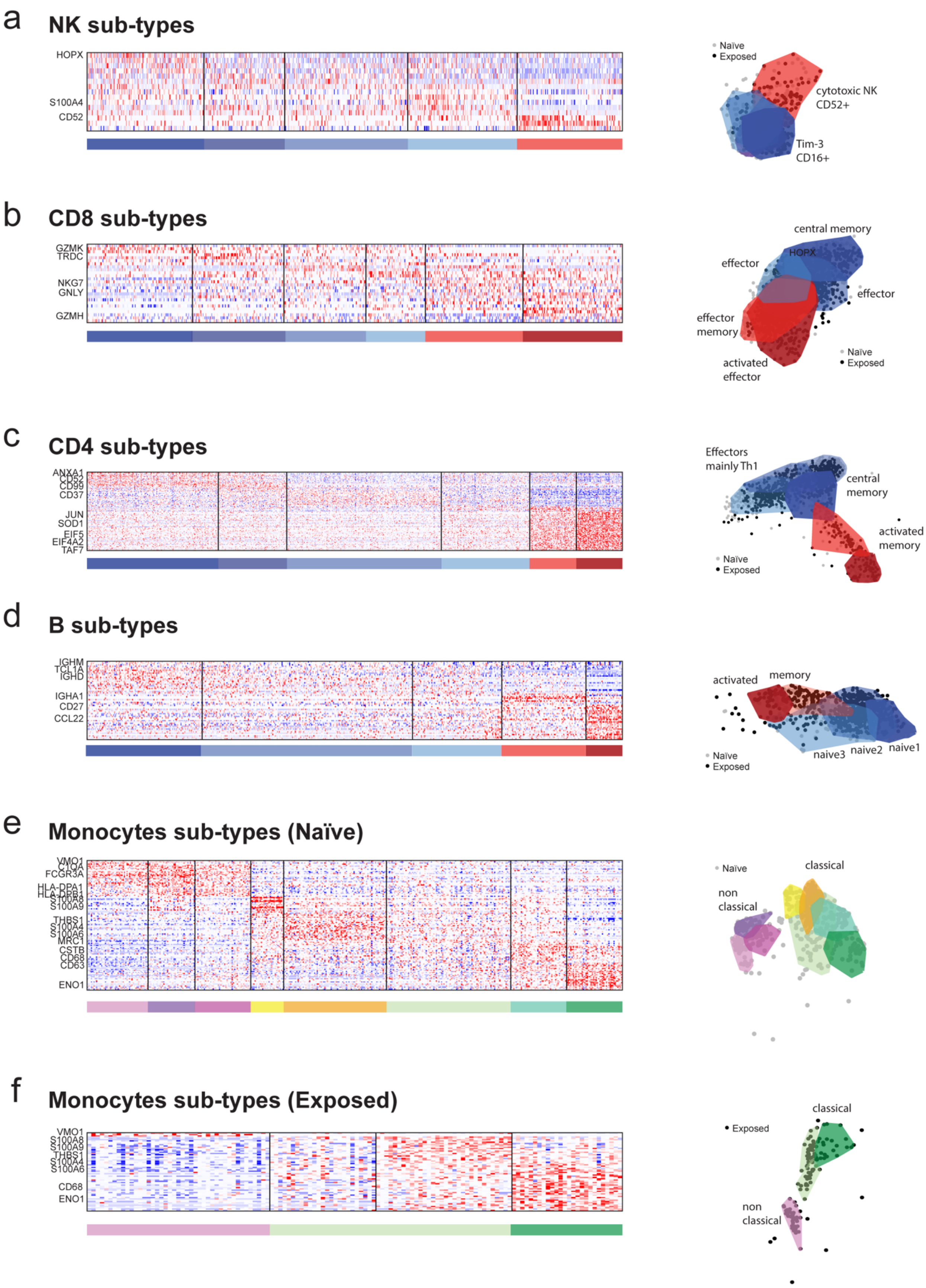
a
NK sub-types
HOPX
S100A4
CD52
Naïve
Exposed
cytotoxic NK
CD52+
Tim-3
CD16+
b
CD8 sub-types
GZMK
TRDC
NKG7
GNLY
GZMH
central memory
HOPX
effector
effector
effector
memory
activated
effector
Naïve
Exposed
c
CD4 sub-types
ANXA1
CD52
CD99
CD37
JUN
SOD1
EIF5
EIF4A2
TAF7
Effectors
mainly Th1
central
memory
activated
memory
Naïve
Exposed
d
B sub-types
IGHM
TCL1A
IGHD
IGHA1
CD27
CCL22
activated
memory
Naïve
Exposed
naive3
naive2
naive1
e
Monocytes sub-types (Naïve)
VMO1
C1QA
FCGR3A
HLA-DPA1
HLA-DPB1
S100A8
S100A9
THBS1
S100A4
S100A6
MRC1
CSTB
CD68
CD63
ENO1
Naïve
classical
non
classical
f
Monocytes sub-types (Exposed)
VMO1
S100A8
S100A9
THBS1
S100A4
S100A6
CD68
ENO1
Exposed
classical
non
classical

Supplementary Figure 7. Intrinsic cell sub-type 'fingerprints' of human PBMCs before and after *ex vivo Salmonella* infection by scRNA-seq

a-f Expression matrix of the genes which are significantly differentially expressed between the various sub-types of each cell type, as indicated in the figure (0.001% FDR level and minimal fold change of 1.4fold). Cell sub-types identity and infection-induced cell state was inferred from these signatures; selected genes which characterize each sub-type are indicated to the left. To the right of each matrix presented is a part of the KNN-graph from fig. 1c, with the naive and exposed cells relevant to the indicated cell type (correspond to fig. 2b; and same color-code).

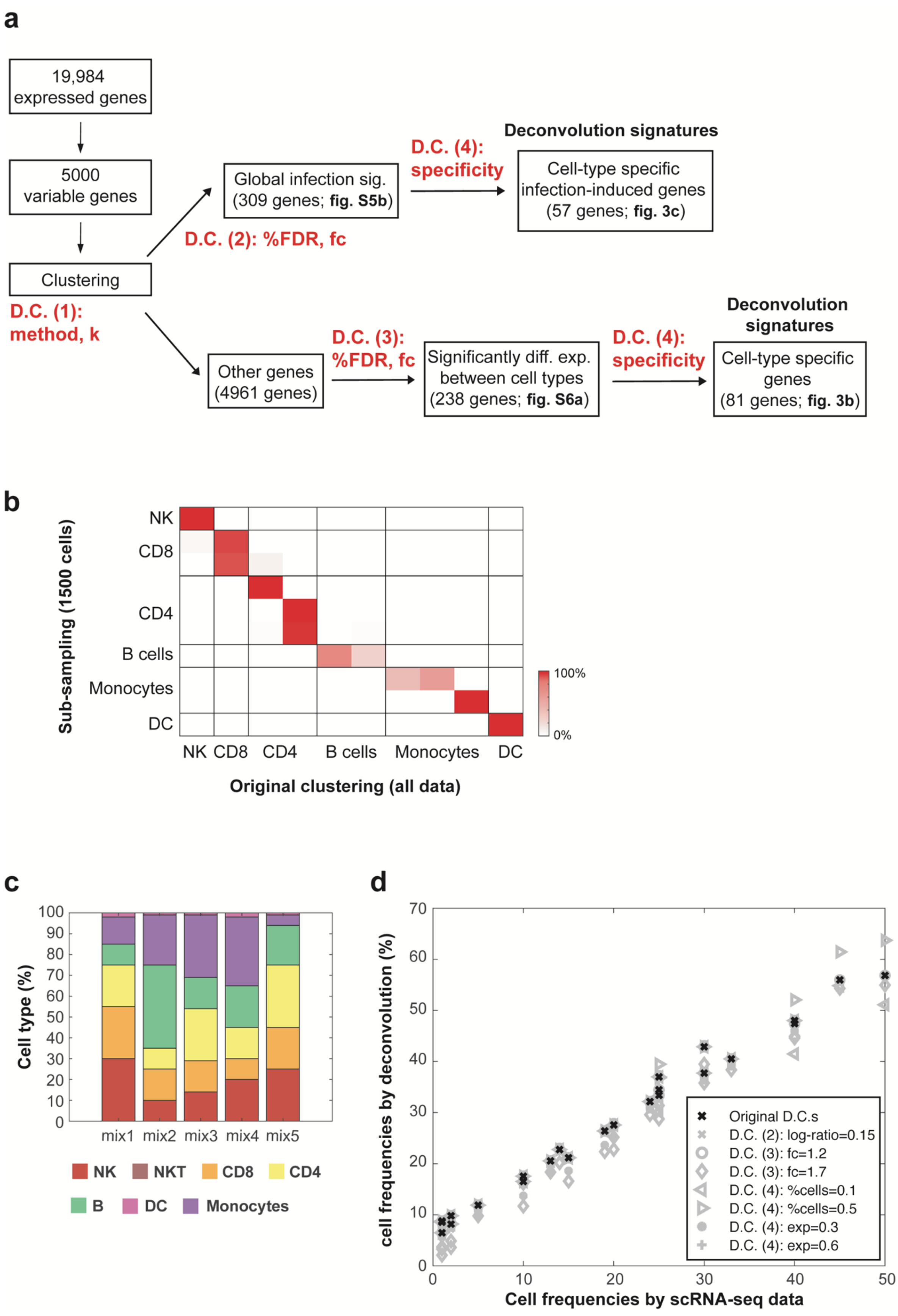

a
19,984 expressed genes
5000 variable genes
Clustering
D.C. (1): method, k
D.C. (2): %FDR, fc
Global infection sig. (309 genes; fig. S5b)
D.C. (4): specificity
Deconvolution signatures
Cell-type specific infection-induced genes (57 genes; fig. 3c)
Other genes (4961 genes)
D.C. (3): %FDR, fc
Significantly diff. exp. between cell types (238 genes; fig. S6a)
D.C. (4): specificity
Deconvolution signatures
Cell-type specific genes (81 genes; fig. 3b)
b
Sub-sampling (1500 cells)
NK
CD8
CD4
B cells
Monocytes
DC
NK CD8 CD4 B cells Monocytes DC
Original clustering (all data)
100%
0%
c
Cell type (%)
100 90 80 70 60 50 40 30 20 10 0
mix1 mix2 mix3 mix4 mix5
NK NKT CD8 CD4
B DC Monocytes
d
cell frequencies by deconvolution (%)
70 60 50 40 30 20 10 0
0 10 20 30 40 50
Cell frequencies by scRNA-seq data
Original D.C.s
D.C. (2): log-ratio=0.15
D.C. (3): fc=1.2
D.C. (3): fc=1.7
D.C. (4): %cells=0.1
D.C. (4): %cells=0.5
D.C. (4): exp=0.3
D.C. (4): exp=0.6

Supplementary Figure 8. Pipeline of the single-cell RNA-seq and deconvolution analysis with robustness analysis all design choices.

a Workflow of the pipeline of the single-cell RNA-seq data which was performed in order to generate the cell-type specific signatures of our deconvolution algorithm. Design choices (D.C.) that were used in our pipeline are highlighted in red (fc; fold change). **b** Comparison of the original clustering of the data (3515 naïve cells; x-axis) relative to sub-sampling of the data (randomly selected 1500 cells; y-axis). Presented is a representative sub-sampling of the data out of 10 simulations. Sub-sampling of the data did not changed the cell clusters, validating its reliability and robustness. **c** We generated from the single-cell data 'bulk-like' samples by averaging the gene expression levels across different compositions of 1000 cells. Presented are the compositions of 5 such synthetic mixes of cells. **d** Comparison of the actual cellular composition of the samples (x-axis) to the relative abundance of each cell type as inferred by our deconvolution algorithm (y-axis). There is high concordance between the actual compositions and all modified design choices (r-squared values range between 0.97-0.99).

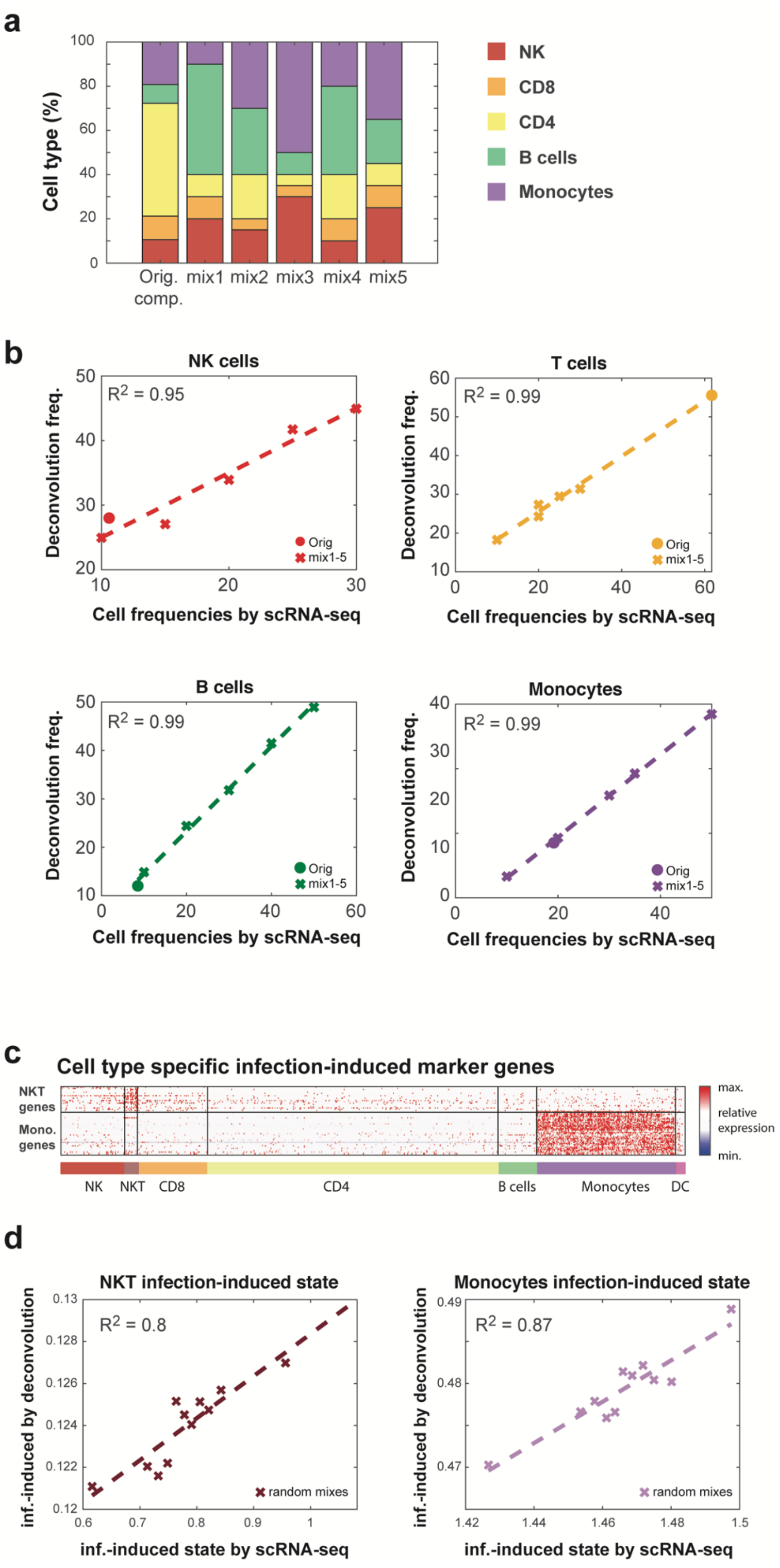
a
Cell type (%)
100
80
60
40
20
0
Orig. comp.
mix1
mix2
mix3
mix4
mix5
NK
CD8
CD4
B cells
Monocytes
b
NK cells
R^2 = 0.95
Deconvolution freq.
Cell frequencies by scRNA-seq
Orig
mix1-5
T cells
R^2 = 0.99
B cells
R^2 = 0.99
Monocytes
R^2 = 0.99
c
Cell type specific infection-induced marker genes
NKT genes
Mono. genes
max.
relative expression
min.
NK
NKT
CD8
CD4
B cells
Monocytes
DC
d
NKT infection-induced state
R^2 = 0.8
inf.-induced by deconvolution
inf.-induced state by scRNA-seq
random mixes
Monocytes infection-induced state
R^2 = 0.87

Supplementary Figure 9. Validation of our deconvolution algorithm in independent datasets.

(a-b) Analysis on public scRNA-seq data from Zheng, G. X. Y. *et al*. **a** Presented is the cellular composition of the original sample and 5 simulated synthetic mixes we have generated in order to test the accuracy and robustness of our algorithm. **b** Comparison between the actual percentage of each cell type (x-axis, from the single-cell data) to the predicted relative abundance by our deconvolution algorithm (y-axis). Our deconvolution algorithm is accurate in an independent individual, with r-squared values between 0.95-0.99, supporting its robustness in an independent sample.

(c-d) Analysis on scRNA-seq data of PBMCs infected *ex vivo* with *Salmonella*, from an independent individual. **c** Expression levels of the NKT and the monocytes infection-induced genes in the new single cell data. The signatures are reproducible and specific in an independent individual, with specific expression of the NKT infection-induced genes only in NKT cells and exclusive expression of the monocytes infection-induced genes from monocytes. **d** We generated 20 synthetic mixes from the single cell data with constant cell-type composition and different infection-induced state of the cells, and transformed it into 'bulk-like' samples. Presented are the comparisons between the inferred infection-induced state of the NKT (left) and the monocytes (right) by our deconvolution algorithm (y-axis) to the infection-induced state of the cells as calculated from the single cell data (x-axis). The inferred infection-induced state by our algorithm is accurate in an independent individual, with high r-squared values (0.8 and 0.87 for NKT and monocytes, respectively), supporting its robustness in an independent sample.

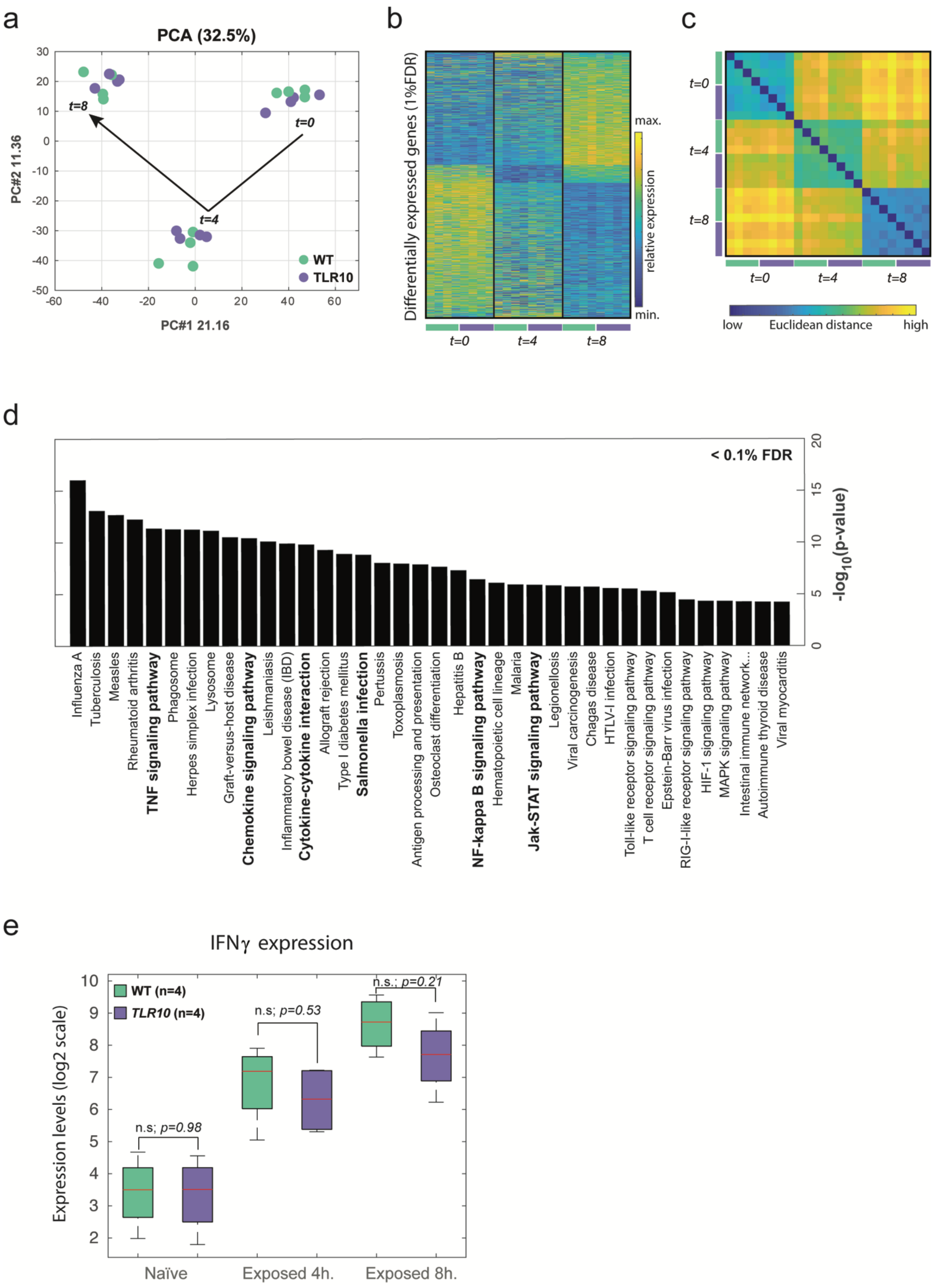
a
PCA (32.5%)
t=8
t=0
t=4
WT
TLR10
PC#2 11.36
PC#1 21.16
b
Differentially expressed genes (1%FDR)
max.
relative expression
min.
t=0
t=4
t=8
c
t=0
t=4
t=8
low
Euclidean distance
high
d
< 0.1% FDR
-log10(p-value)
Influenza A
Tuberculosis
Measles
Rheumatoid arthritis
TNF signaling pathway
Phagosome
Herpes simplex infection
Lysosome
Graft-versus-host disease
Chemokine signaling pathway
Leishmaniasis
Inflammatory bowel disease (IBD)
Cytokine-cytokine interaction
Allograft rejection
Type I diabetes mellitus
Salmonella infection
Pertussis
Toxoplasmosis
Antigen processing and presentation
Osteoclast differentiation
Hepatitis B
NF-kappa B signaling pathway
Hematopoietic cell lineage
Malaria
Jak-STAT signaling pathway
Legionellosis
Viral carcinogenesis
Chagas disease
HTLV-I infection
Toll-like receptor signaling pathway
T cell receptor signaling pathway
Epstein-Barr virus infection
RIG-I-like receptor signaling pathway
HIF-1 signaling pathway
MAPK signaling pathway
Intestinal immune network...
Autoimmune thyroid disease
Viral myocarditis
e
IFNγ expression
WT (n=4)
TLR10 (n=4)
Expression levels (log2 scale)
n.s; p=0.98
n.s; p=0.53
n.s.; p=0.21
Naïve
Exposed 4h.
Exposed 8h.

Supplementary Figure 10. Bulk RNA-seq analysis of WT vs. *TLR10* individuals following *ex vivo Salmonella* infection.

a PCA projection of the bulk RNA-seq samples onto the space of the two leading principal components showed marked differences between infection timepoints, but no differences between WT and *TLR10* individuals. Each dot is a sample (green for WT and purple for *TLR10* individuals), and the time post-infection is indicated next to the dots (t=0, 4 or 8). **b** Heat map for the expression levels of 1834 genes that are significantly differentially expressed following infection in all individuals (1% FDR; colorbar to the right indicates expression level). **c** Distance matrix (corresponding to the expression matrix in **b**) between the 24 bulk RNA-seq samples over the space of the differentially expressed genes following infection (1%FDR), with blue denoting short distance (e.g. similar) and yellow large distance. Green bars indicate WT individuals and purple *TLR10* individuals (t stand for the time post-infection in hours). **d** KEGG-pathway enrichments analysis of the 1834 differentially expressed genes following infection (FDR<0.1%; genes from **b**). **e** Box plot of the expression levels of IFNγ as measured by the bulk RNA-seq data of our 8 individuals (green for WT (n=4) and purple for *TLR10* individuals (n=4)). On each box, the central mark indicates the median, and the box the 25th-75th percentile; whiskers encompass all data points. P-values for the differences in expression between WT and *TLR10* individuals in each time point are indicated at the figure. n.s. = not significant, two samples t-test.

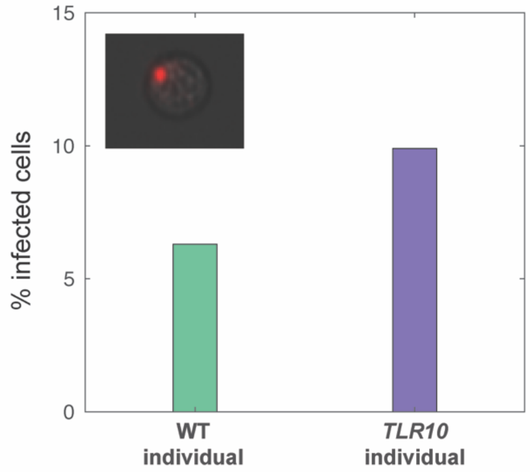

Supplementary Figure 11. Differences in cell-type composition and cell-cell signaling is associated with different intracellular bacterial control by WT and *TLR10* individuals

PBMCs form one WT individual and one *TLR10* individual were infected *ex vivo* with *Salmonella* expressing RFP under a constitutive promoter, and subjected to imaging flow cytometry (ImageStream). Presented are the percentage of infected cells from each sample. Representative image of a cell infected with bacteria (red) is shown at the top.

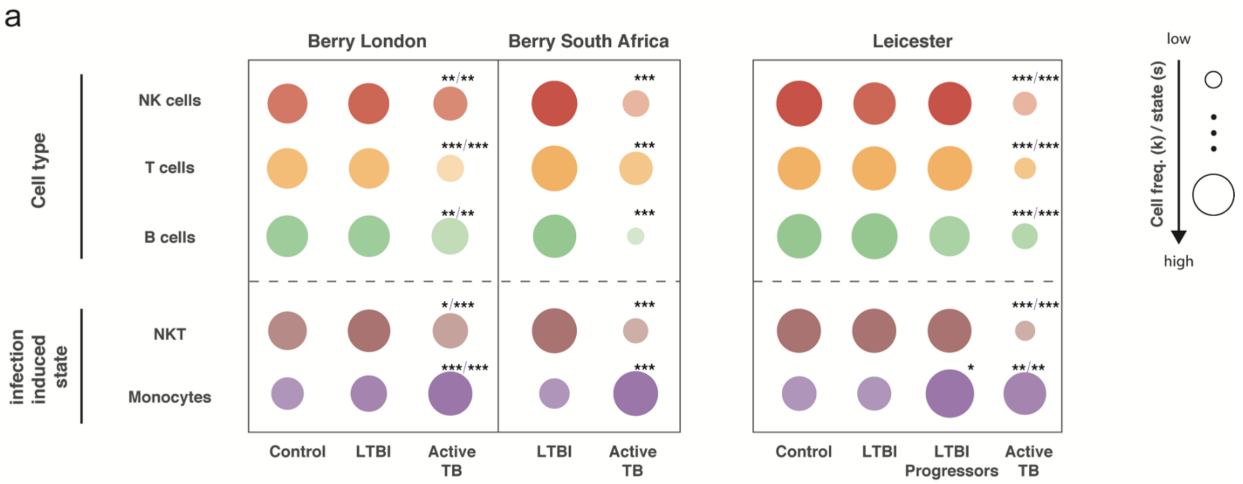

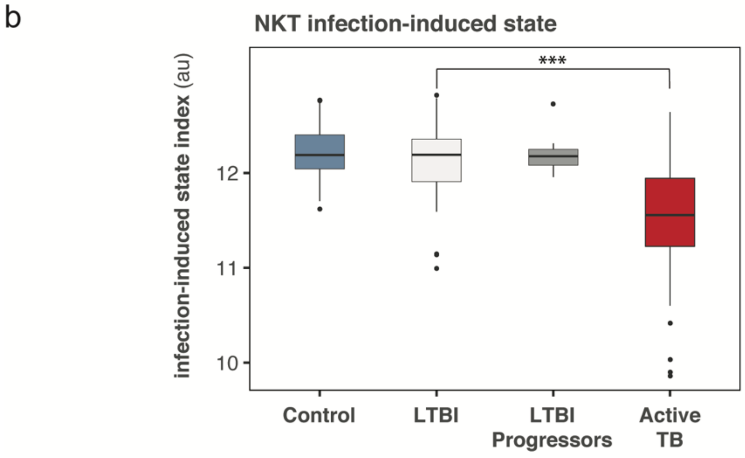

Supplementary Figure 12. Changes in cell-type composition and infection-induced state in the blood of active TB patients.

a Deconvolution of cell-type composition and the infection-induced cell states in the blood of control, LTBI and active TB patients from 3 datasets (Berry London GSE107991, Berry South Africa GSE107992 and Leicester GSE107994). The size of the circles are equivalent to the size of the estimators of the cell-type frequency or infection-induced state, with each row normalized to mean 0 and standard deviation of 1. Colors are as in fig. 1b. For Leicester datasets there is a separation between LTBI individuals who remained healthy (LTBI), and these who developed to active TB later (progressors). * p<0.05, ** p<0.01, ***p<0.001, two samples t-test. p-values are indicated next to the circles: Berry London (controls vs. active TB)/(LTBI vs. active TB), Berry South Africa (LTBI vs. active TB), Leicester progressors (LTBI vs. progressors), and Leicester active TB (controls vs. active TB)/(LTBI vs. active TB). **b** Box plot of the NKT

infection-induced state in the blood of control individuals (blue; n=50), LTBI individuals who remained healthy (light gray; n=49), LTBI individuals who developed active TB (progressors; dark gray, n=8) and active TB patients (red; n=53) from Leicester dataset at baseline. On each box, the central mark indicates the median, and the box the 25th-75th percentile; whiskers encompass interquartile range. *** p-value <0.0001, two samples t-test. Values are inferred from the bulk measurement using our deconvolution algorithms, estimators of cell infection-induced state index are in arbitrary units (au).
