# Supplementary material for: Predicting bacterial infection outcomes using single cell RNA-sequencing analysis of human immune cells
Source: Nat Commun. 2019 Jul 22;10:3266. doi: 10.1038/s41467-019-11257-y (PMC6646406; doi:10.1038/s41467-019-11257-y)
Supplement: Supplementary file 2 — Description of Additional Supplementary Files [file 41467_2019_11257_MOESM2_ESM.pdf]

Description of Additional Supplementary Files

File name: Supplementary Data 1

Description: Cluster-specific genes for naïve and exposed samples

File name: Supplementary Data 2

Description: Repertoire of immune cell sub-types from the scRNA-seq data

File name: Supplementary Data 3

Description: GO-term enrichment analysis for our global infection signature

File name: Supplementary Data 4

Description: Evaluation of the algorithm robustness to D.C. 2

File name: Supplementary Data 5

Description: Evaluation of the algorithm robustness to D.C. 3

File name: Supplementary Data 6

Description: Evaluation of the algorithm robustness to D.C. 4

File name: Supplementary Data 7

Description: Expression of the monocytes infection-induced signature in isolated monocytes, neutrophils, PBMCs and whole-blood cells of healthy controls and TB patients

File name: Supplementary Data 8

Description: Subset of the monocytes infection-induced signature which is highly specific to monocytes and up-regulated in TB patients
