# Supplementary material for: Predicting bacterial infection outcomes using single cell RNA-sequencing analysis of human immune cells
Source: Nat Commun. 2019 Jul 22;10:3266. doi: 10.1038/s41467-019-11257-y (PMC6646406; doi:10.1038/s41467-019-11257-y)
Supplement: Supplementary file 11 — Reporting Summary [file 41467_2019_11257_MOESM11_ESM.pdf]

nature research

Corresponding author(s): Roi Avraham

Reporting Summary

Nature Research wishes to improve the reproducibility of the work that we publish. This form provides structure for consistency and transparency in reporting. For further information on Nature Research policies, see Authors & Referees and the Editorial Policy Checklist.

Statistical parameters

When statistical analyses are reported, confirm that the following items are present in the relevant location (e.g. figure legend, table legend, main text, or Methods section).

n/a	Confirmed	
☐	☒	The exact sample size (n) for each experimental group/condition, given as a discrete number and unit of measurement
☐	☒	An indication of whether measurements were taken from distinct samples or whether the same sample was measured repeatedly
☐	☒	The statistical test(s) used AND whether they are one- or two-sided<br>*Only common tests should be described solely by name; describe more complex techniques in the Methods section.*
☐	☒	A description of all covariates tested
☐	☒	A description of any assumptions or corrections, such as tests of normality and adjustment for multiple comparisons
☐	☒	A full description of the statistics including central tendency (e.g. means) or other basic estimates (e.g. regression coefficient) AND variation (e.g. standard deviation) or associated estimates of uncertainty (e.g. confidence intervals)
☐	☒	For null hypothesis testing, the test statistic (e.g. F, t, r) with confidence intervals, effect sizes, degrees of freedom and P value noted<br>*Give P values as exact values whenever suitable.*
☒	☐	For Bayesian analysis, information on the choice of priors and Markov chain Monte Carlo settings
☒	☐	For hierarchical and complex designs, identification of the appropriate level for tests and full reporting of outcomes
☒	☐	Estimates of effect sizes (e.g. Cohen's d, Pearson's r), indicating how they were calculated
☐	☒	Clearly defined error bars<br>*State explicitly what error bars represent (e.g. SD, SE, CI)*

Our web collection on statistics for biologists may be useful.

Software and code

Policy information about availability of computer code

Data collection	No software was used to collect the data (publicly available datasets of TB patients were downloaded from GEO)
Data analysis	For pre-processing of the single cell RNA-seq data we used the Cell Ranger Single-Cell Software Suite (https://support.10xgenomics.com/single-cell-gene-expression/software/pipelines/latest/what-is-cell-ranger)<br><br>For pre-processing of the bulk RNA-seq data we used the cel-seq pipeline (https://github.com/yanailab/CEL-Seq-pipeline)<br><br>To sort gene expression matrix of the bulk RNA-seq data we used the SPIN algorithm (Tsafrir, D. et al. Sorting points into neighborhoods (SPIN): data analysis and visualization by ordering distance matrices. Bioinforma. Oxf. Engl. 21, 2301–2308 (2005))<br><br>For Go-terms and KEGG pathway enrichment we used DAVID (https://david.ncifcrf.gov)<br><br>Custom code was used for the analysis of the single cell and bulk RNA-seq data<br><br>Custom code was used for the deconvolution algorithm (publicly available at: https://github.com/noabossel/Dynamic-deconvolution-algorithm)

For manuscripts utilizing custom algorithms or software that are central to the research but not yet described in published literature, software must be made available to editors/reviewers upon request. We strongly encourage code deposition in a community repository (e.g. GitHub). See the Nature Research guidelines for submitting code & software for further information.

Data

Policy information about availability of data

All manuscripts must include a data availability statement. This statement should provide the following information, where applicable:

- Accession codes, unique identifiers, or web links for publicly available datasets
- A list of figures that have associated raw data
- A description of any restrictions on data availability

The data was uploaded to GEO under the SuperSeries GSE122084.

Field-specific reporting

Please select the best fit for your research. If you are not sure, read the appropriate sections before making your selection.

☒ Life sciences ☐ Behavioural & social sciences ☐ Ecological, evolutionary & environmental sciences

For a reference copy of the document with all sections, see nature.com/authors/policies/ReportingSummary-flat.pdf

Life sciences study design

All studies must disclose on these points even when the disclosure is negative.

Sample size

WT/TLR10 data: We recruited 8 healthy individuals to this study, 4 WT and 4 with TLR10 polymorphism. Sample size was determined according to the availability of samples with the required polymorphism.
TB cohorts: we used 3 published cohorts of TB patients; we used all available data.

Data exclusions

For the bulk RNA-seq data (WT/TLR10) we had 3 replicates for each sample. For 3 samples, one of their repeats were with less than 100K exonic reads and therefore were excluded due to low coverage. Thus, for 3 samples, only 2 replicates were used instead of 3.
This is indicated in the methods section.

Replication

All CFU experiments were done with at least 3 replicates samples and with at least two independent experiments.
All bulk RNA-seq data were done with 2-4 replicate samples.

Randomization

Randomization was not relevant for the WT/TLR10 dataset since individuals were allocated to groups based on their TLR10 polymorphism (bearing or not TLR10 polymorphism).

Blinding

Blinding was not relevant for data collection, since individuals were allocated to groups based on their TLR10 polymorphism.
For CFU experiments, the researchers were blinded to the category of the individual (WT/TLR10).

Reporting for specific materials, systems and methods

Materials & experimental systems

n/a	Involved in the study
☒	☐ Unique biological materials
☐	☒ Antibodies
☒	☐ Eukaryotic cell lines
☒	☐ Palaeontology
☒	☐ Animals and other organisms
☐	☒ Human research participants

Methods

n/a	Involved in the study
☒	☐ ChIP-seq
☐	☒ Flow cytometry
☒	☐ MRI-based neuroimaging

Antibodies

Antibodies used

anti-hIFN-g antibodies (Clone # 25718, R&D systems, cat. MAB285, lot KW041804A)
IgG2A isotype control (Clone # 20102, R&D systems, cat. MAB003, lot MV0917051)
APC/Cy7 anti-human CD14 antibodies (Clone # M5E2, BioLegend, cat. 301820, lot B251038)
PE/Cy7 anti-human CD19 (Clone # HIB19, BioLegend, cat. 302216, lot B242979)
APC anti-human CD56 (Clone # NCAM16.2, BD Biosciences, cat. 341027, lot 8253651)
PE anti-human CD3 (Clone # UCHT1, BioLegend, cat. 300408, lot B238975)

Validation

anti-hIFN-g antibodies: https://www.rndsystems.com/products/human-ifn-gamma-antibody-25718_mab285
IgG2A isotype control: https://www.rndsystems.com/products/mouse-igg2a-isotype-control_mab003
APC/Cy7 anti-human CD14 antibodies: https://www.biolegend.com/fr-fr/products/apc-cy7-anti-human-cd14-antibody-3293
PE/Cy7 anti-human CD19 antibodies: https://www.biolegend.com/en-us/products/pe-cy7-anti-human-cd19-antibody-1911
APC anti-human CD56 antibodies: https://www.citeab.com/antibodies/2414653-341027-cd56-apc
PE anti-human CD3: https://www.citeab.com/antibodies/520421-300408-pe-anti-human-cd3-antibody-ucht1-monoclonal

Human research participants

Policy information about studies involving human research participants

Population characteristics

WT/TLR10 dataset: participants were categorized based on genotypic information, their TLR10 locus polymorphism (bearing or not TLR10 polymorphism, N241H (rs11096957))

Recruitment

Participants were recruited at the Radboud University Medical Center at the Netherlands. All participants were healthy with no self-selection biases.

Flow Cytometry

Plots

Confirm that:

☒ The axis labels state the marker and fluorochrome used (e.g. CD4-FITC).

☒ The axis scales are clearly visible. Include numbers along axes only for bottom left plot of group (a 'group' is an analysis of identical markers).

☒ All plots are contour plots with outliers or pseudocolor plots.

☒ A numerical value for number of cells or percentage (with statistics) is provided.

Methodology

Sample preparation

Cells were washed with PBS and suspended with FACS buffer (20% FBS and 1mm EDTA in PBS), then stained with the relevant antibodies for 30min in 4°c under dark conditions. After washings with cold PBS, cells were resuspended with FACS buffer, stained with live/dead staining SYTOX blue, and analyzed in FACSAria™ III flow cytometer (BD Biosciences)

Instrument

FACSAria™ III flow cytometer (BD Biosciences)

Software

Diva software

Cell population abundance

The gating strategy for sorting: monocytes were sorted according to FSC/ SSC localization, NKT cells were sorted according to CD3+ CD56+ positive cells.

Gating strategy

"cells" gate: according to FSC-A/SSC-A, without cells derbies
"Singles" gate from "cells" gate was determined according to FSC-A/FCS-H, gating the cells on the diagonal
"Live cells" from "singles" population were determined according to Sytox blue staining, gating the population that was not stained
"RFP-positive" cells from "live" population were gated according to RFP intensity, higher that 10^3 considered as positive
"CD14+" cells from "live" population were gated according to APC/Cy7 intensity, higher that 10^3 considered as positive
"RFP-positive" cells from "CD14+" population were gated according to RFP intensity, higher that 10^3 considered as positive
"CD19+" cells from "live" population were gated according to PE/Cy7 intensity, higher that 10^3 considered as positive
"CD3+" cells from "live" population were gated according to PE intensity, higher that 10^3 considered as positive
"CD56+" cells from "live" population were gated according to APC intensity, higher that 10^3 considered as positive
NKT cells were gated as CD3+CD56+ double positive.
Monocytes were sorted according to FSC/ SSC localization
NKT cells were sorted according to CD3+ CD56+ positive cells

☒ Tick this box to confirm that a figure exemplifying the gating strategy is provided in the Supplementary Information.
